# Resveratrol and Cardiovascular Diseases

**DOI:** 10.3390/nu8050250

**Published:** 2016-05-02

**Authors:** Dominique Bonnefont-Rousselot

**Affiliations:** 1 Department of Biochemistry, Faculty of Pharmacy, Paris Descartes University, Sorbonne Paris Cité, Paris 75006, France; dominique.rousselot@aphp.fr; Tel.: +33-1-42-16-20-58; Fax: +33-1-42-16-20-33; 2 Department of Metabolic Biochemistry, Pitié-Salpêtrière-Charles Foix Hospital (AP-HP), Paris 75013, France; 3 Inserm UMR_S 1166 ICAN, UPMC, La Pitié Hospital, Paris 75013, France

**Keywords:** antioxidant, atherosclerosis, clinical, heart failure, hypertension, inflammation, myocardial infarction, preclinical, resveratrol

## Abstract

The increased incidence of cardiovascular diseases (CVDs) has stimulated research for substances that could improve cardiovascular health. Among them, resveratrol (RES), a polyphenolic compound notably present in grapes and red wine, has been involved in the “French paradox”. RES is known for its antioxidant and anti-inflammatory properties and for its ability to upregulate endothelial NO synthase (eNOS). RES was able to scavenge ^•^OH/O_2_^•−^ and peroxyl radicals, which can limit the lipid peroxidation processes. Moreover, in bovine aortic endothelial cells (BAEC) under glucose-induced oxidative stress, RES restored the activity of dimethylargininedimethylaminohydrolase (DDAH), an enzyme that degrades an endogenous inhibitor of eNOS named asymmetric dimethylarginine (ADMA). Thus, RES could improve ^•^NO availability and decrease the endothelial dysfunction observed in diabetes. Preclinical studies have made it possible to identify molecular targets (SIRT-1, AMPK, Nrf2, NFκB…); however, there are limited human clinical trials, and difficulties in the interpretation of results arise from the use of high-dose RES supplements in research studies, whereas low RES concentrations are present in red wine. The discussions on potential beneficial effects of RES in CVDs (atherosclerosis, hypertension, stroke, myocardial infarction, heart failure) should compare the results of preclinical studies with those of clinical trials.

## 1. Introduction

The increased incidence of cardiovascular diseases (CVDs) (atherosclerosis, hypertension, stroke, ischemic heart diseases, heart failure, *etc.*) will lead to an expected worldwide number of CVD-related deaths of more than 23.6 million by 2030 [1]. Resveratrol (RES) is a non-flavonoid polyphenolic compound that is a stilbene derivative. It is a phytoalexin produced by plants, and is notably present in grapes and red wine. It could play a potential protective role against CVDs, and be involved in the “French paradox” characterized by the low incidence of CVDs in the French population despite a high intake of saturated fats, in association with moderate red wine consumption [2]. Even if a combination of polyphenols, rather than RES alone, may account for this paradox [3,4], RES is a good candidate, owing to its protective action of vascular walls towards oxidation, inflammation, platelet oxidation and thrombus formation [4]. However, some difficulties in interpreting results can arise from the use of high-dose supplements of RES that are often used for research studies, whereas there are low concentrations of RES in red wine [5,6]. Moreover, despite the exponential increase in published studies on RES, few are related to human clinical trials [3]. Long-term well-controlled trials are thus needed to confirm its beneficial effect on CVDs.

## 2. Resveratrol as an Antioxidant Able to Improve Nitric Oxide Availability *in Vitro*

One of the cardioprotective mechanisms of RES is due to its ability to upregulate eNOS [7,8], which favors nitric oxide-mediated vasodilation. As noted below, *in vitro* experiments under conditions mimicking diabetes, *i.e.*, in the presence of high glucose concentrations, have indirectly shown a potential benefit of RES on endothelial function, by improving the bioavailability of nitric oxide (^•^NO).

Diabetes is a well-known CV risk factor. It is characterized by a chronic hyperglycemia, micro- and macrovascular complications including an accelerated atherosclerosis, lipid accumulation in the arterial intima, chronic inflammation and oxidative stress [9]. This is concomitant with an endothelial dysfunction, a proinflammatory phenotype, an intracellular oxidative stress, and consequent perturbations of the ^•^NO pathway [10]. The physiological roles of ^•^NO consist in improving the vasodilation and decreasing platelet aggregation, leukocyte recruitment and proliferation of smooth muscle cells [11], which is in favor of an inhibition of atherosclerosis formation and progression. RES could exhibit beneficial properties both as an antioxidant and as a regulator of ^•^NO metabolism. Note that RES exists in two isomeric forms, *trans*- and *cis*-RES [7], with a conversion of the isomer *trans* into the *cis* form under UV radiation, the *trans*-RES being the principal biologically active form.

As regards oxidative stress, previous experiments have shown the ability of RES to directly scavenge hydroxyl (^•^OH) and superoxide (O_2_^•−^) radicals generated by water gamma radiolysis, a method that allows us to quantitatively manage free radical production. This scavenging action has been evidenced by simultaneously monitoring the radical-induced disappearance of *trans*-RES and the concomitant formation of RES oxidation products identified by mass spectrometry (essentially piceatannol (trans-3,5,3′,4′-tetrahydroxystilbene) and 3,5-dihydroxybenzoic acid under our experimental conditions) [12,13]. The radical scavenging properties of trans-RES were also involved in the inhibition of lipid peroxidation. Indeed, RES exhibited a protective effect against linoleate micelle oxidation initiated by radiolysis-generated ^•^OH radicals. Piceatannol nevertheless exhibited a better antioxidant effect, probably due to the presence of an additional hydroxyl group; under these conditions, a more efficient scavenging of lipid peroxyl radicals ^•^LOO was observed with piceatannol than with RES [14].

Metabolism of ^•^NO depends on a balance between two phenomena, *i.e.*, formation of asymmetric dimethylarginine (ADMA) from protein catabolism via methyltranferase protein II (PRMT), and activity of dimethylarginine dimethylaminohydrolase (DDAH), an enzyme that is involved in ADMA catabolism. Indeed, ADMA negatively controls the production of ^•^NO from l-Arginine by eNOS, by amplifying eNOS uncoupling. The enzyme isoform referred to as DDAH2 degrades ADMA into citrulline, which suppresses eNOS inhibition. Since DDAH2 is inhibited by oxidative stress, ADMA is no more catabolized under these conditions and accumulates in the cell, which leads to an important eNOS inhibition and an endothelial dysfunction [10,15]. Conditions of oxidative stress also results in eNOS uncoupling, especially related to the decreased availability of tetrahydrobiopterine (eNOS cofactor), which triggers a production of O_2_^•−^ instead of ^•^NO [16]. With the aim to know if RES could prevent ADMA formation under high glucose-mediated oxidative stress conditions, bovine aortic endothelial cells (BAEC) were incubated with or without RES at 0.1, 0.5, 1 or 10 µM in the presence of 25 mM glucose for 24 h. The results showed that oxidative stress induced by this incubation with a high glucose concentration (conditions that mimic the pathological conditions of the diabetes) led to an inhibition of DDAH activity, and that RES restored DDAH activity in a dose-dependent manner. RES restored DDAH expression only under conditions of oxidative stress, not under basal conditions [17]. No effect of RES was observed on the expression or the activity of arginase (enzyme that catalyzes arginine into ornithine and urea), whereas a relationship has been reported between an increased arginase activity and the development of endothelial dysfunction [18,19]. The sensitivity of DDAH to oxidative stress induced by high glucose concentrations could be explained by the presence of cysteine residues in the active site of DDAH [20]. Concomitantly with the inhibition of DDAH, the oxidative stress conditions induced by a 24 hr-incubation with 25 mM glucose increased ADMA intracellular production, the latter being lower in the presence of RES [17]. This led to conclude that RES regulated DDAH activity after glucose-induced oxidative stress in BAEC. Note that a similar observation on the DDAH/ADMA pathway has been reported by Yuan *et al.* [21] in a glucose-induced endothelial cell senescence model, via sirtuin activation.

**Figure 1:**
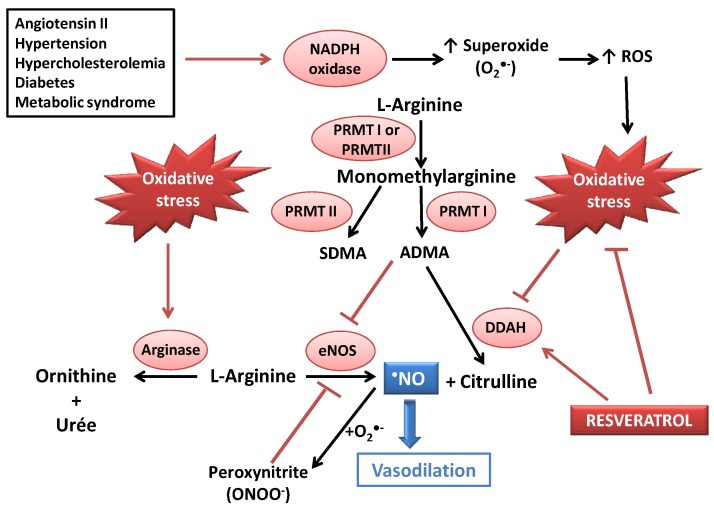
Deleterious effects of oxidative stress on ^•^NO bioavailability, and potential beneficial action of resveratrol (RES) as an antioxidant and as a modulator of the dimethylaminohydroase (DDAH)/asymmetric dimethylarginine (ADMA) pathway. CVDs could lead to increased ADMA concentrations (due to DDAH inhibition) and activation of NADPH oxidase, leading to oxidative stress. ADMA and peroxynitrite both induce eNOS decoupling, resulting in a decreased ^•^NO availability. RES could act as an antioxidant agent (radical scavenging and stimulation of antioxidant defenses) and as an activator of DDAH. ADMA: asymmetric dimethylarginine; DDAH: dimethylarginine dimethylaminohydrolase; eNOS: endothelial NO synthase; PRMT: protein methyltransferase; ROS: Reactive oxygen species; SDMA: symmetric dimethylarginine.

As previously observed regarding radical scavenging, a higher efficiency of piceatannol has been observed in this ability to restore ^•^NO bioavailability. On the whole, the beneficial effects of RES either by direct oxygen-derived radical scavenging or by increased ^•^NO bioavailability *in vitro* could promise potential benefits in CVDs [16] (Figure 1).

## 3. Health Benefits of Resveratrol against Cardiovascular Diseases: Examples of Preclinical and Clinical Studies

Several preclinical studies on animal models have highlighted beneficial effects of RES on CVDs [22], supported by the identification of multiple molecular targets for RES (*i.e*., silent information regulator 2/sirtuin 1 (SIRT-1), AMP-activated protein kinase (AMPK), nuclear factor (erythroid-derived 2)-like 2 (Nrf2), nuclear factor-kappa B (NF-κB), *etc.*) [23]. Micro-RNAs could play a key role as regulators in cardiac functions, as shown by miRNA profiles that were different in patients with coronary artery disease and in healthy subjects. Thus, miR-126, miR-27, miR-92a and miR-155 were lowered in these patients, whereas myocardial-derived miRNAs such as miR-133 and miR-208 were increased [24]. Few miRNAs are suggested as regulators involved in cardioprotection, and heart preconditioning by RES could result in up or down regulation of these miRNAs [25], as discussed below, especially regarding myocardial ischemia. Nevertheless, there are a limited number of human clinical trials, with sometimes inconsistent findings [26]. The potential beneficial effects of RES on CVD observed in preclinical studies thus need to be discussed in the light of these clinical studies in the main CVDs, *i.e.*, atherosclerosis, hypertension, stroke, myocardial ischemia and heart failure.

### 3.1. Anti-Atherosclerotic Effects of RES

Atherosclerosis predominantly affects the intimal layer of the arterial vessel wall. It is characterized by the deposition of extracellular lipids, the proliferation and migration of local smooth muscle cells, and a chronic inflammation. It leads to luminal narrowing and/or thrombus formation, resulting in clinical events such as coronary artery disease, peripheral arterial disease or stroke [27]. Due to the involvement of lipids, especially low density lipoproteins (LDLs), in the atherosclerotic process, it could be of interest to improve the lipid profile. Some preclinical studies have shown that RES could modify this profile, notably by decreasing plasma triglyceride and LDL-cholesterol levels, and by increasing HDL-cholesterol [28]. As reported by Cho *et al.* [29], RES could also potentiate the hypocholesterolemiant action of pravastatin, by down-regulating the 3-hydroxy-3-methyl-glutaryl-CoAreductase (HMG-CoA reductase), an enzyme that intervenes in the first steps of cholesterol biosynthesis. Besides, RES could increase the expression of the LDL receptors (LDL-R) in hepatocytes *in vitro* [30], thereby contributing to further decrease blood LDL-cholesterol levels. In addition, the antioxidant properties of RES resulted in a decrease of LDL oxidation (process directly involved in atherogenesis [31]), an induction of several endogenous antioxidant systems [32], and anti-inflammatory properties [33]. The inhibition of smooth muscle cell migration also participates to the antiatherogenic properties of RES [34]. All these properties show that RES acts on the major factors involved in the atherosclerotic process.

**Figure 2:**
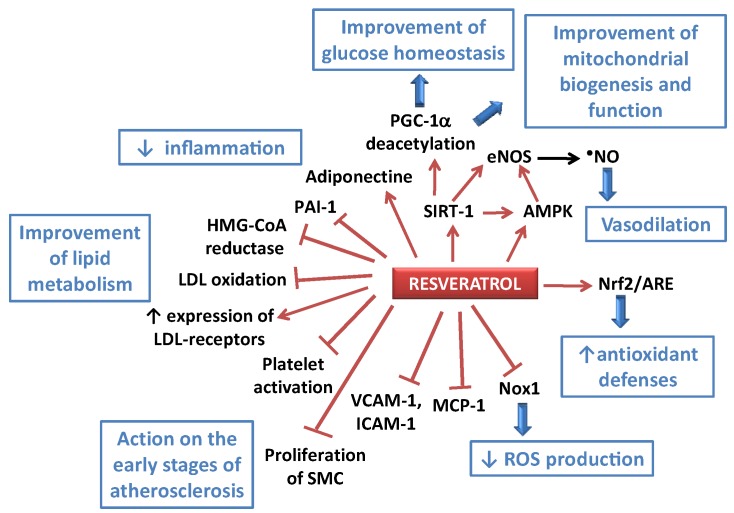
Some of the potential effects of resveratrol (RES) towards atherogenesis and impaired metabolism. AMPK: AMP-activated protein kinase; ARE: antioxidant response element; eNOS: endothelial NO synthase; HMG-CoA reductase: 3-hydroxy-3-methyl-glutaryl-CoA reductase; ICAM-1: intercellular adhesion molecule-1; LDL: low density lipoprotein; MCP-1: monocyte chemotactic protein-1; NF-κB: nuclear factor-kappa B; Nox1: NADPH oxidase 1; Nrf2: nuclear factor (erythroid-derived 2)-like 2; PAI-1: plasminogen activator inhibitor-1; PGC-1αperoxisome proliferator-activated receptor-γ co-activator 1α; ROS: reactive oxygen species; SIRT-1: silent information regulator 2/sirtuin 1; SMC: smooth muscle cells; VCAM-1: vascular cell adhesion molecule-1.

Accordingly, several potential targets related to the beneficial effects of RES in CVDs have been highlighted. RES especially activates SIRT-1 (a class III histone deacetylase), eNOS, Nrf2 and antioxidant response element (ARE), and decreases TNFα production. The global action of RES thus results in a decrease of endothelial apoptosis, endothelial activation and vascular inflammation, and improves the endothelial function [35]. Actions of RES in the first steps of the atherogenic process have been observed. Indeed, RES has been shown to decrease the expression of adhesion molecules (intercellular adhesion molecule-1, ICAM-1, and vascular cell adhesion molecule-1, VCAM-1) via inhibition of NF-κB pathway activation [36]. RES could also counteract the formation of foam cells by inhibiting NADPH oxidase 1 expression and the production of monocyte chemotactic protein-1 (MCP-1) in macrophages treated with lipopolysaccharide, via an action on the Akt and forkhead box O3a (FoxO3a) pathways [37]. RES effects on inflammation could involve the modification of the expression of miRNAs that can be anti-inflammatory (e.g., miR-663) or pro-inflammatory (e.g., miR-155) [38]. As regards anti-inflammatory actions of RES, it has also been shown to activate Nrf2 and suppress proinflammatory cytokine production in cardiomyocytes, thereby alleviating endotoxin-induced myocardial injury in mice, which could constitute a potential way to prevent sepsis-induced cardiomyopathy [39]. Another effect of RES that could contribute to its anti-atherogenic effect is the inhibition of the migration and proliferation of vascular smooth muscle cells [34,40] (Figure 2).

On the other hand, the clinical studies showed either an absence of effect on the lipid profile, as reported in a meta-analysis of seven clinical trials [41], or beneficial effects. As an example, treatment with 250–1000 mg RES/day lowered LDL-cholesterol in patients with type 2 diabetes [42]. Similarly, treatment with RES decreased plasma triglycerides in healthy obese men (150 mg RES/day) [43] and in healthy adult smokers (500 mg RES/day) [44]. In patients at high CV risk under statin treatment for primary prevention, RES (350 mg/day of RES-enriched grape extract containing 8 mg RES) led to a 20% decrease of oxidized LDL and 4.5% decrease in LDL-cholesterol [45]. Nevertheless, the anti-atherosclerotic effect of RES should not be limited to an effect on serum lipid profile and needs to be proven by large clinical trials.

### 3.2. Anti-Hypertensive Effects of RES

Hypertension constitutes a major risk factor for CVDs [46]. Anti-hypertensive effects of RES have been reported in several animal models of hypertension, after treatment by 10 to 320 mg RES/kg body weight/day, for 14 days to 10 weeks, depending on the studies [5]. It is noteworthy that relatively low doses of RES (5–10 mg/kg/day) significantly lowered blood pressure in animal models associating hypertension with insulin resistance [47], which could suggest that RES would be more efficient in patients with diabetes or metabolic syndrome. In several studies, RES was administered prior to the development of hypertension. For instance, Dolinsky *et al.* [48] observed that a high dose of RES attenuated high pressure and prevented cardiac hypertrophy in two hypertensive animal models, namely spontaneously hypertensive rats and angiotensin-II infused mice. A few studies have shown the ability of RES to reverse cardiac hypertrophy and contractile dysfunction, two structural and functional abnormalities associated with hypertension [49,50]. In a recent study [51], RES alone has been shown to be ineffective at reducing blood pressure in 28 weeks old spontaneously hypertensive rats, but a combination of RES with hydralazine (a blood pressure lowering agent) was more effective than hydralazine alone in improving CV parameters. It can be noted that in all studies RES treatment was of short-term duration.

The mechanisms involved in the antihypertensive properties of RES can be endothelium-dependent, with the implication of AMPK (a regulator of energy metabolism), SIRT-1 and Nrf2 [5]. This results in a vasodilation via an improved availability of ^•^NO, in relation to increased expression and activity of eNOS [7,8], and this property is associated with the antioxidant properties of RES [52]. RES-induced activation of SIRT-1 increased both expression and activity of eNOS [53]. RES is also able to activate AMPK thereby increasing ^•^NO production [48]. Thus, RES has been shown to improve flow-mediated vasodilation in several animal models [54,55]. Besides, endothelium-independent mechanisms have also been reported [5], such as an inhibition of vascular smooth muscle cells contractility, via AMPK activation, leading to an inhibition of angiotensin II (AngII)-induced phosphorylation of myosin phosphatase-targeting subunit 1 and myosin light chain [56]. In addition, the authors showed that RES inhibited the AngII-induced aorta contractions, an effect that was abolished by AMPK inhibition. Accordingly, daily treatment with RES decreased hypertension in an experimental model of AngII-induced hypertensive mice [56].

As regards clinical studies, a meta-analysis of six randomized controlled trials (including 247 subjects) showed that high doses of RES (≥150 mg/day) significantly decreased blood pressure, while lower doses had no effect [57]. The decrease in blood pressure is often associated with an improvement of metabolic parameters, which constitutes a confounding factor. It is noteworthy that the antihypertensive action of RES, as evaluated by the increase in acetylcholine-evoked vasorelaxation, was more pronounced if RES was administered to hypertensive and dyslipidemic subjects [58], which could be related to what has been previously observed in animal models [47].

### 3.3. Protective Effect of RES in Stroke

A protective effect of RES against ischemic stroke have been reported in adult rats and related to a protection of endothelial function [59]. Accordingly, treatment of cell culture with RES improved cell viability against oxygen and glucose deprivation (conditions that mimic an *in vitro* “ischemia”). This endothelial protection would be dependent on SIRT-1, as shown by the suppression of the effect when SIRT-1 was inhibited with sirtinol. In relation to this effect, RES protected endothelial cells, decreased brain damage and inflammation, and preserved blood brain barrier function [59]. Similarly, RES decreased the infarct size in a rat model of focal cerebral ischemia [60]. Treatment with RES succeeded in preventing diabetes-induced impairment of eNOS-dependent vasorelaxation in cerebral arterioles of type 1 diabetic rats [61], which could be of interest for the treatment of cerebrovascular dysfunction in diabetic patients. In adition, neuroprotective effects of RES have been described [62], and mechanisms of this neuroprotection were proposed in a recent study conducted on rats [63]; the authors showed that RES inhibited phosphodiesterases and regulated the cAMP/AMPK/SIRT1 pathway, which reduced ATP energy consumption during ischemia. The levels of ATP, phospho-AMPK, SIRT1, and cAMP were thus increased by RES.

Unfortunately, no investigation has been conducted in stroke patients. Nevertheless, RES has been shown to increase cerebral blood flow in healthy adult subjects [64,65], which could participate to potential beneficial effects of this molecule in stroke patients. Similarly, RES has also been proposed to enhance cerebrovascular perfusion in postmenopausal women [66].

### 3.4. Effects of RES on Myocardial Ischemia

Some mechanisms have been proposed to explain the effects of RES under conditions of myocardial ischemia. They include an inhibition of platelet aggregation, a preconditioning of muscle tissue to ischemia-reperfusion (IR), and a potential regeneration of tissue in the infarcted area [67]. Preclinical studies showed that pretreatment with RES resulted in a protection against the deleterious effects of myocardial reperfusion after ischemia [68,69,70], especially by decreasing infarct size and reducing arrhythmia. The efficiency of these pretreatments suggests that RES could precondition the myocardium to fight against IR injury. This preconditioning could act by switching death signals due to IR into survival signals, through the activation of Akt and Bcl-2 [71]. RES effects are not limited to IR models, since they are also observed under conditions of permanent ischemia. Thus, cardioprotection has been reported after RES pretreatment, in infarction models obtained by left anterior descending coronary artery ligation; RES induced a reduction of infarct size and an improvement of cardiac function [72,73,74]. Similarly, in a porcine model of metabolic syndrome and chronic myocardial ischemia, RES supplementation resulted in significant improvements of body mass indices, serum cholesterol and C-reactive protein concentrations, glucose tolerance and myocardial metabolism [75]. However, this protective effect depended on the dose and duration of RES treatment: e.g., treatment of rats with 2.5 mg RES/kg/day for 10 days induced protection, whereas 25 or 100 mg RES/kg/day did not [76]. Besides, some studies have reported a protective effect when RES was given after myocardial infarction, which is closer to the pathological conditions and consequently more relevant than a pretreatment for the clinical practice, since RES could thus be administered to reverse the CV complications associated with ischemic heart disease [77,78,79].

RES confers its anti-ischemic effects through multiple mechanisms, *i.e.*, activation of AMPK, eNOS and vascular endothelial growth factor (VEGF), and by decreasing oxidative stress [5]. Indeed, activation of AMPK and/or SIRT-1 by RES has been shown to induce autophagy in the cardiomyocyte, which is considered as a protective mechanism. For instance, pretreatment of H9c2 cells (*i.e.*, a rat myocardium-derived cardiac myoblast cell line) with RES before hypoxia decreased cell death during the reoxygenation, with a mechanism dependent on AMPK and autophagy. Autophagy is a cellular process by which damaged components are removed, and it might be cardioprotective when well regulated. This induction of autophagy has also been observed in rat myocardium, through the activation of mammalian target of rapamycin (mTOR)-Rictor (mTOR complex 2, mTORC2) survival pathway [76]. In connection with the autophagy, RES has been shown to inhibit fraktalkin, a protein that inhibits the RES-induced increase of autophagy in cardiomyocytes [77].In a swine model of metabolic syndrome with induction of chronic myocardial ischemia, Sabe *et al.* [80] recently showed that RES regulated autophagy signaling and they confirmed that this might be a mechanism by which RES exerts its cardioprotection. Another key mechanism involved in the beneficial effect of RES in myocardial infarction is the ability of RES to increase the levels of ^•^NO, which is a potent vasodilator that improves tissue perfusion. For instance, pretreatment with RES increased the expression of the endothelial and inducible NOS isoforms (eNOS and iNOS, respectively) in the ischemic myocardium of rats, associated with a significantly enhanced expression of VEGF and its tyrosine kinase receptor Flk-1; this allows to confer a proangiogenic effect of RES in the infarcted myocardium [73]. Other authors reported an increased expression of iNOS in rat heart after IR, but no change in eNOS or neuronal NOS (nNOS) expression. Nevertheless, under RES preconditioning, expression of eNOS and nNOS was greatly upregulated, but without any induction of iNOS. These results could suggest that the effect of RES on nNOS and iNOS expression were at the transcriptional level, whereas RES could act on eNOS expression via a reduced protein turnover [68]. By using NOS inhibitors, Hung *et al.* [68] concluded that iNOS and eNOS, but not nNOS, may be involved in the protective effect of RES in myocardial infarction after IR.

The antioxidant properties of RES also play a role in its protective effects towards IR damage. Indeed, reperfusion after ischemia induces a high production of reactive oxygen species (ROS), responsible for tissue damage, and RES is able to induce antioxidant defense mechanisms and to limit lipid peroxidation under these conditions [81]. In addition, in a swine model with chronically ischemic myocardium, RES induced an increase in peroxiredoxin 2, a protein known to scavenge free radicals [82]. Closely related to oxidative stress, inflammation is also a key player in myocardial IR injury. A recent study [83] has been performed on rats with myocardial IR, with administration of RES at the onset of reperfusion. The results suggested that the Natch domain, Leucine-rich repeat and PYD-containing protein 3 (NALP3) inflammasome was activated during the process, with secretion of the inflammatory cytokines IL-1β and IL-18 mediating the cascade inflammatory response. The authors showed that RES may protect the myocardium against IR injury by inhibiting the expression and activation of the NALP3 inflammasome, and that the resulting decrease of the inflammatory response may be involved in the cardioprotective mechanisms of RES.

Recent research has highlighted the role of microRNAs (miRNAs) in the cardioprotective effects of RES in ischemic heart. These endogenous small non-coding RNA molecules contain less than 25 nucleotides, and act in RNA silencing and post-transcriptional regulation of gene expression. RES could thus protect the heart towards ischemia by restoring the upregulation or down-regulation of gene expression induced by *ex vivo* IR in rats given 5 mg RES/kg/day for 21 days [84]. Under these IR conditions, 25 miRNAs were either up or down-regulated, and pretreatment of animals with RES led to reverse the up or down regulation. RES thus modulated miR-21, miR-20b, miR-27a, miR-9. Among these, there was a high upregulation of miR-21 expression in basal level controls with RES, which was considerably lowered in IR; miR-21 has been shown to regulate the ERK-MAPK signaling pathway in cardiac fibroblasts, which is involved in cardiac structure and remodeling [85]; miR-21 also regulated fibroblast metalloprotease 2 in a murine myocardial infarction model, with a specific localization in the infarct region of the IR heart [86]. The vascular endothelial growth factor (VEGF) has been reported to be modulated by miR-20b via the hypoxia-inducible factor 1-alpha (HIF1α) in response to hypoxia [87], whereas FoxO1 was regulated by miR-27a in cancer cells [88], and SIRT-1 by miR-9 in stem cells [89]. The reported role of RES in cardioprotection could thus be mediated by its action on miRNAs, as suggested by the unique signature of miRNA expression induced by RES treatment [25]. Among the miRNAs regulated by myocardial infarction, there are members of the miR-29 family, which have been reported to be down-regulated in the heart, in the region adjacent to the infarct; these miRNA target mRNAs encoding proteins that are involved in fibrosis, so that down-regulation of miR-29 would enhance the fibrotic response; consequently, down-regulation of miR-29 with modified antisense oligonucleotides, referred to as anti-miRs could induce the expression of collagens, whereas over-expression of miR-29 in fibroblasts would reduce collagen expression, which could constitute a potential therapeutic way to regulate cardiac fibrosis [90]. In a model of cold exposure-induced cardiac hypertrophy in mice, RES has been shown to inhibit the increase of miR-328 involved in cardiac hypertrophy, thereby limiting ultrastructure damage and weakened heart functions [91].

In contrast with the multiple preclinical studies, few clinical trials have been conducted to investigate the effects of RES in patients with ischemic heart disease. In patients with stable angina or acute coronary syndrome, a 3-arm parallel, randomized, triple-blind, placebo-controlled trial, has tested a one-year treatment with either placebo (*n* = 25), conventional grape extract (GE) (*n* = 25), or GE containing RES (*n* = 25) [92]. The treatment consisted of 350 mg/day (containing 8 mg RES) for 6 months, then 700 mg/day (16 mg RES) for the next 6 months. The results showed that the combination GE plus RES led to an increase in adiponectin, a decrease in plasminogen activator inhibitor-1 (PAI-1), and lowered blood lipids. Nevertheless, the latter effect was also observed in the GE group, which suggests that it could be related to other components than RES present in GE. Another study has been conducted in patients with myocardial infarction (MI), in order to investigate the role of RES in secondary prevention [93]. This was a 3-month double-blind, placebo-controlled, randomized trial, including 40 post-infarction Caucasian patients (26 men and 14 women), treated either by a placebo or by 10 mg RES/day associated with a standard medication. RES decreased LDL-cholesterol, improved systolic function and endothelial function, and decreased platelet aggregation. The authors conclude that, in association with a standard medication, RES treatment of post-MI patients may be a promising way to decrease the risk of secondary MI; indeed, RES improved heart function, endothelial function, red blood cell deformability, and decreased serum LDL-cholesterol level and platelet aggregation. However, the poor bioavailability of RES still remains a subject of discussion for the efficiency of RES after oral administration [94]. A moderately high dose (100 mg) of RES given *per os* would thus result in almost the same blood concentration as a very low dose (10 µM) administered intravenously [67]. Militaru *et al.* [95] evaluated the effects of a 60-day oral supplementation with RES, calcium fructoborate (CF), and their combination in patients with stable angina pectoris, in a randomized, double-blinded, active-controlled, parallel clinical trial conducted in three groups of subjects. The combination of these two substances was based on the fact that CF, a nutritional supplement that contains calcium, fructose and boron in a sugar-borate ester form, would stabilize RES degradation in the digestive tract and increase its anti-inflammatory properties. The combination of RES and CF decreased the number of angina episodes and thereby improved the quality of life of the patients; the N-terminal prohormone of brain natriuretic peptide (NT-proBNP), a biomarker of heart failure, was significantly lowered by RES (59.7% decrease after 60 days) and by CF (52.6% after 60 days), their combination being the most effective (65.5% after 60 days). Another double-blind, placebo controlled trial has been conducted in 40 post-infarction Caucasian patients randomized into two groups [93]; in the group receiving 10 mg RES daily for three months, the authors reported an improved left ventricle diastolic function, a better endothelial function, lowered LDL-cholesterol levels and protection against red blood cell deformability and platelet aggregation.

### 3.5. Effects of RES on Heart Failure

In heart failure, the heart is not able to provide the tissues with enough oxygen and nutrients. This situation can arise from several situations, such as MI, hypertension, cardiomyopathies, *etc.* Cardiac remodeling appears as a response to heart failure, but this can also contribute to the pathogenesis of this dysfunction [67].

In most preclinical studies, administration of RES was performed prior to the development of heart failure and this allowed prevention of cardiac hypertrophy and improvement of cardiac function [74,78]. However, some studies evaluated the effects of RES administered after the induction of heart failure, which could be of higher relevance in clinical practice. For instance, Kanamori *et al.* [79] induced MI in mice via left coronary artery occlusion, and RES was given four weeks after the surgery at a time when left ventricle ejection fraction (LVEF) was significantly reduced (39% *vs.* 74% in sham animals). Two weeks of treatment with RES (50 mg/kg/day) improved LVEF from 39% to 47%, probably due to a decrease of the size of the infarct and a reversal of the maladaptive remodeling. A recent study [96] has evaluated the effects of RES in a model of hypertension in mice; administration of RES (10 mg/kg/day) after surgery reduced pathological cardiac remodeling and dysfunction; moreover, it decreased oxidative stress, inflammation, fibrosis and apoptosis.

RES interferes with several processes implicated in the pathophysiology of cardiac hypertrophy and heart failure [5]. The mechanisms involved include a decrease in oxidative stress, evidenced by an enhanced expression of the antioxidant mitochondrial enzyme Mn-superoxide dismutase (SOD2) [97], an increase in the antioxidant glutathione levels, an activation of eNOS, an inhibition of protein synthesis (AMPK activation and Akt inhibition [98]), an improvement of calcium cycling (activation of the sarcoplasmic/endoplasmic reticulum Ca-ATPase 2a (SERCA2) expression via SIRT-1 [99]), and an inhibition of hypertrophic gene expression. RES also induces autophagy through both AMPK and SIRT-1 pathways [5]. Nevertheless, at low concentrations (1 µM), the mechanisms by which RES inhibited left ventricle hypertrophy appeared to be AMPK-independent in rat cardiomyocytes, while they were AMPK-dependent at higher concentrations (50 µM), and RES seemed to be essentially effective in pathological cardiac hypertrophy [100]. Nevertheless, controversies exist regarding the induction of SIRT-1 by RES [99], and there is a debate on whether activation of SIRT-1 is the cause or the consequence of RES action.

Unfortunately, no publication is available on the effect of RES in patients with heart failure. Patients with stable coronary artery disease treated with 10 mg RES/day exhibited an improvement of diastolic function (and low increase in systolic function) after MI [93]. This suggests that RES could be of interest in the treatment of MI-induced cardiac dysfunction.

## 4. Discussion

Clinical studies regarding treatment with RES are not as promising as the preclinical findings as regards the beneficial effects of RES on CVDs. This could be partly related to the low availability of RES, due to its rapid metabolism [94]. Nevertheless, the concept of the “RES paradox” (biological effects of RES despite low plasma concentrations) has been proposed [101]. This paradox could thus be related to a possible action of RES metabolites [102]. Other polyphenols or micronutrients of the Mediterranean diet could also present a synergistic effect of RES, thereby contributing to this paradox [4].

Bioavailability of RES has been extensively studied, since RES undergoes a rapid conjugation in intestine and liver (formation of glucuronides and sulfates) that limits oral availability [103,104]. Thus, after oral administration to healthy subjects (0.36 mg/kg body weight), only approximately 2 µM RES were detected in plasma within 30 min. [105], which was far below the EC_50_ values (5–100 µM) determined for pharmacologic effects [103]. This extensive *in vivo* metabolism tends to suggest that metabolites could have pharmacologic activity [106]. Cottart *et al.* [104] reported in their literature review that very low plasma concentrations of free RES were detectable, if any, after consumption of wine or juices; when RES was administered at a dose of about 25 mg (that is, the dose approximately provided by wine consumption, with a RES concentration in wine up to 5.8 mg/L), the RES plasma concentration ranged from 1 to 5 ng/mL, whereas doses up to 5 g resulted in plasma concentrations up to 530 ng/mL. A systematic pharmacokinetic study has been conducted by Boocock *et al.* [107] after oral administration of RES (single doses of 0.5, 1, 2.5, or 5 g) in healthy volunteers (10 subjects per dose level); peak plasma concentrations of RES were from 73 to 539 ng/mL (0.3 to 2.4 µM, respectively) after intake of 0.5 and 5 g RES, respectively, with a slight rebound after 5–6 h related to the enterohepatic cycle. The plasma concentrations of the main metabolites (RES-3-O-sulfate and monogluccuronides) were higher, e.g., 1135–4294 ng/mL (3.7–14 µM) for RES-3-O-sulfate. Several studies aimed to improve the availability of RES, either by modifying the structural determinant or by using RES oligomers or galenic forms such as calcium-pectinated beads [108], polymeric micelles [109], self-emulsifying systems [110] or nanoparticles [111]. Nevertheless, it should be borne in mind that low doses of RES could be more effective than higher doses; dose-response studies are therefore needed [112], together with development of new technologies such as microencapsulation [113] or nanoparticles [114] to better target tissues.

The dose of RES that should be used in clinical studies effectively remains a key question. The effects of a moderate alcohol intake have been evaluated [115] in a study including 224 adults given a Mediterranean diet: 83 controls drank water, 68 white wine, 73 red wine (as a 150-mL drink with dinner for 2 years). Although containing 4- to 13-fold higher RES levels than white wine, red wine was less efficient in decreasing glycemia and insulin resistance, which again suggested that other components could intervene in the beneficial CV effects of RES. Besides, high amounts of RES could be cytotoxic, so that RES could exert beneficial effects at lower doses and cytotoxic effects at higher doses [116], which defines hormesis (dose-response relationship beneficial at low doses and detrimental at higher doses, resulting in a J-shaped or an inverted U-shaped dose-response curve). Cytotoxicity of RES could also be related to a prooxidant effect, although this property has rather been observed with higher hydroxylated RES derivatives that were able to form quinones, and compounds possessing ortho-hydroxy groups were stronger cytotoxic agents than compounds without such a structure [117].

In addition, it is noteworthy that RES possibly interacts with drugs, notably CVD medications. The question is of interest, because a lot of RES food supplements are available, with sometimes much higher dosages than the natural amounts of RES. Indeed, RES has been shown to inhibit drug metabolizing enzymes, such as cytochromes P450 (CYP), enzymes that constitute a superfamily of oxidases responsible for phase-I oxidative metabolism of xenobiotics. RES has especially been shown to inhibit CYP1A2, CYP1B1 and CYP1A1 *in vitro* [118], although a weak induction of CYP1A2 has been reported by Chow *et al.* [119]. Induction of drug metabolizing enzymes such as uridine diphosphate glucuronyosyl transferase (UGT) A1 has also been reported [120]. These effects of RES could lead to safety problems by modifying circulating and/or tissue concentrations of co-administered drugs. Concentrations required for *in vitro* CYP inhibition were in the 1 to 100 µM range, and IC_50_ values of RES for CYP3A4 activity were between 1 and 5 µM, *i.e.*, lower concentrations than for the other CYP [121]. Apart from systemic CYP inhibition, intestinal interactions should also be considered [121]. When RES is given as a few milligrams daily (which is close to the amounts of RES contained in red wine, *i.e.*, an average of 1.9 ± 1.7 mg/L or 8.3 ± 7.4 µM *trans*-RES), there is a low risk of critical interactions with the intestinal metabolism of co-administered drugs [121]. The modulation of drug metabolizing enzyme activity by RES (1 g/day for four weeks) has been studied in healthy subjects, and has not shown any effect of UGT1A1 or glutathione-S-transferase activity, but has evidenced a loss of activity in CYP [119]. Besides, no inhibition of CYP1A2, CYP2C19, CYP2D6 and CYP3A4 has been observed *in vitro* with the main RES metabolite formed in human plasma, *i.e.*, RES-3-sulfate [122]. Although well tolerated by healthy volunteers, high doses of RES (around 1 g/day or above) could lead to interactions with co-administered drugs, these interactions resulting from inhibition of intestinal CYP3A4 and/or P-glycoprotein (Pgp), which is an energy-dependent efflux pump that exports its substrates out of the cell [121]. Nevertheless, it is noteworthy that very high plasma levels of RES (aglycone) could only be reached after administration of high doses and/or specific drug formulations (e.g., micronisation), and the risk of drug interactions essentially involved CYP3A4; this is also to be discussed as a function of the high protein binding of RES, which limits the risk of interaction [121]. The question of a RES-induced inhibition of CYP3A4 leads to possible interactions with medications used in CVDs, especially statins (HMG-CoA reductase inhibitors), calcium channel blockers and anti-arrhythmic agents [123]. In addition, the natural blood pressure-lowering and anti-coagulant effects of RES may cause possible interaction with blood pressure, anti-platelet, and anticoagulant medication, and with nonsteroidal anti-inflammatory drugs such as aspirin and ibuprofen.

Finally, the recent editorial published by Visioli [124] highlights the inconclusive results of some human studies, partly due to often small groups of patients, short-term studies, and differences in the protocols used. According to this author [124], only the study conducted by Tomé-Carnero *et al.* [125] was reasonably-sized and suggested anti-inflammatory activities in CV patients. Human clinical trials are sparse when compared to the huge body of cellular and preclinical studies, and it seems difficult to translate the voluminous and sometimes conflicting preclinical data into clinical evidence [126], since concerns related to the dose and the availability of RES remain [124]. Twenty-six publications are thus reported as clinical trials in PubMed, using “resveratrol” and “cardiovascular disease” as keywords (Table 1). Of these publications, five were not clinical trials with RES supplementation, since RES was only used *ex vivo* [127,128] or *in vitro* [129], one study did not use RES but crocin [130] and another did not aim to report beneficial effect of RES in patients but only to analyze urinary total RES metabolites as a biomarker of moderate wine consumption [131]. It also appears that controversial data have emerged from recent studies, with no beneficial effects of RES supplementation in non-alcoholic fatty liver disease (NAFLD) patients [132], or in overweight and slightly obese patients [133]. No association of the RES levels achieved with a Western diet with inflammation markers or CVDs has been observed [134]. Similarly, no effect of RES has been reported on arterial stiffness, endothelial function, blood pressure, or metabolic variables in nonobese men and women, perhaps in relation to a lack of availability of RES [135].

However, it is important to note that CVDs constitute an important source of comorbidity in diabetic and obese subjects, and RES could be of potential interest in these populations. In this context, RES has been shown to improve heart function in streptozotocin-induced diabetes in mice [99] and rats [136]. In animal models of type 2 diabetes, the diastolic function was also better after RES treatment [137]. RES has been shown to improve glucose homeostasis in mice via a SIRT-1-mediated deacetylation of peroxisome proliferator-activated receptor-γ co-activator 1α (PGC-1 α) [138], although some controversies have emerged regarding the induction of SIRT-1 by RES [99]. Activation of SIRT-1 by RES has also be disputed because there is questioning about the original study reporting its ability to activate SIRT-1 in an artificial substrate-based fluorescent assay [139]. The data reported by Pacholec *et al.* [140] have provided evidence that RES was not a direct SIRT-1 activator. RES effects could be mediated by other pathways: RES can thus activate AMPK through an upstream kinase LKB1 (liver kinase B1) in the absence of SIRT-1 [139]. Because SIRT-1 is most active in times of energy demand, when NAD^+^ amounts or the NAD^+^/NADH ratio are the highest, SIRT-1-mediated deacetylation and activation of PGC-1α also constitute an important response of the cell to increase mitochondrial biogenesis and function [141]. As an example, treatment of cultured coronary arterial endothelial cells by 10 µM RES for 48 h enhanced the mitochondrial mass [142]. In a diet-induced obesity model in rodents also exhibiting early stages of type 2 diabetes, Louis *et al.* [143] and Qin *et al.* [144] showed that RES treatment improved diastolic function and decreased cardiac hypertrophy. A recent study reported that RES administration could inhibit cold exposure-induced cardiac hypertrophy in mice; the hearts of these mice showed the upregulation of hypertrophy-related miR-328, and RES treatment (100 mg/kg/day) for eight weeks inhibited the increase of miR-328, and had a suppressive action of apoptosis of myocardium via inhibition of Bax and caspase-3 activation [91]. Interestingly, in addition to cardioprotective effects related to its antioxidant and anti-hypertrophic properties, RES could improve insulin sensitivity in animal models of diabetes [145] and metabolic syndrome [146], in relation to anti-diabetic effects [147]. In line with these protective effects, a meta-analysis of 11 randomized clinical trials has shown that RES improved insulin sensitivity and glycemic control in diabetic patients [148]. Mitochondrial dysfunction is considered to play a key role in the development of insulin resistance (IR), and the imbalance between mitochondrial oxidative stress and antioxidant levels is the critical factor in mitochondrial damage [149]. In this context, RES has been shown to prevent mitochondrial dysfunction in a rat model of type 2 diabetes (namely, Zucker diabetic fatty (ZDF) rats) [150]. Similarly, in high-fat diet (HFD)-induced IR rats, an 8-week RES treatment protected rats against diet induced IR, increased SIRT-1 (and SIRT-3) expression, mtDNA, and mitochondrial biogenesis; moreover, mitochondrial antioxidant enzymes were enhanced, which decreased oxidative stress [149]. In coronary artery disease patients with type 2 diabetes and hypertension, RES treatment (grape extract containing 8 mg RES for 12 months) lowered the expression of proinflammatory cytokines by acting via inflammation-related miRNAs (*i.e*., miR-21, miR-181b, miR-663, miR-30c2, miR-155 and miR-34a), which supports an immunomodulatory effect of RES in these patients [125].

## 5. Conclusions

Taking into account the beneficial effects of RES on hypertension, obesity, inflammation, diabetes and dyslipidemia, RES could constitute an interesting pharmacological approach for the treatment of metabolic syndrome, which is associated with an increased risk of CVD development. Nevertheless, among the points to discuss for the interpretation of preclinical and clinical studies, not only the poor bioavailability and the dose of RES are critical, but also the length of RES treatment and the best time for initiating it (most studies showed effectiveness of RES when it was administered for short periods and as a pretreatment) [67]. Larger controlled human clinical trials are thus needed to investigate these points and to study the effects of long-term RES supplementation.

## Figures and Tables

**Table 1:** Publications referred to as clinical trials using RES in CVDs. (1 mg/L RES = 4.38 µmol/L RES).

Authors	Date	Place	Aim of the Study and Population Studied	Dose of RES	Summary of Main Findings	Side Effects
Faghihzadeh *et al.* [132]	2015	Iran	Evaluation of the effects of RES supplementation on CV risk factors in 55 patients with non alcoholic fatty liver disease (NAFLD), in a randomised double-blinded placebo-controlled clinical trial	Supplementation with a 500-mg RES capsule or a placebo capsule for 12 weeks	Supplementation with RES did not have any beneficial effect on anthropometric measurements, insulin resistance markers, lipid profile and blood pressure; however, it reduced alanine aminotransferase and hepatic steatosis in patients with NAFLD.	None reported
Amadio *et al.* [129]	2015	Italy	Study of the effect of prostaglandin E2 (PGE2) induced by cigarette smoke on tissue factor (TF) expression and activity in endothelial cells. No CVD.	10 µM RES only used in vitro as SIRT-1 activator	(1) *In vivo*: positive correlation between PGE2 levels and TF antigen and activity in human smokers; similar findings in mice; (2) *in vitro*: exogenous or endogenous PGE2 up-regulated TF expression and activity via the EP1/SIRT1 pathway in endothelial cells. Cell treatment with exogenous PGI2 prevented the inhibition of SIRT1 and the induction of TF mediated by PGE2.	Not applicable (*in vitro* use of RES as SIRT-1 activator)
Van der Made *et al.* [133]	2015	The Netherlands	Randomized, placebo-controlled crossover study conducted in 45 overweight and slightly obese men (*n* = 25) and women (*n* = 20), with a mean age of 61 ± 7 years. Study of the effect of RES on apoA-I concentrations, and on other markers of lipid and lipoprotein metabolism, glucose metabolism, and markers of inflammation and endothelial function.	Subjects received in random order RES (150 mg per day) or placebo capsules for 4 weeks, separated by a 4-week wash-out period.	RES did not change metabolic risk markers related to CV health in overweight and slightly obese men and women. Effects on glucose metabolism not significant.	None reported
Hobbs *et al.* [151]	2014	USA	Effects of a multi-ingredient supplement featuring red yeast rice in patients with no CVD history or symptoms other than elevated blood lipids.	Study conducted in 19 hypercholesterolemic patients, to evaluate the effects of the supplement alone (in patients with trigly cerides <140 mg/dL) or associated with ω3-polyunsaturated fatty acids and vitamin E (in patients with triglycerides >140 mg/dL, receiving 1384 mg of ω-3 daily). The supplement contained a blend of red yeast rice, biofla-vonoids, polycosanol, 525 mg natural ω-3 fatty acids, RES, coenzyme Q10, folic acid, niacin, B6, B12, and black pepper.	The supplement decreased total cholesterol and LDL-cholesterol significantly, and addition of an ω-3 supplement also non significantly decreased triglycerides in the subgroup with triglycerides >140 mg/dL. Very small size of the group and RES associated with other components.	None reported
Semba *et al.* [134]	2014	USA	Prospective cohort study, the Invecchiare in Chianti (InCHIANTI) Study (“Aging in the Chianti Region”), conducted (1998–2009) in a population-based sample of 783 community-dwelling men and women ≥ 65 years, to determine whether RES levels achieved with diet were associated with inflammation, cancer, cardiovascular disease, and mortality in humans	783 subjects had 24-h urine samples available for measurements of RES (no data on the dietary amount of RES)	Total urinary RES metabolite concentration was not associated with inflammatory markers, CVD or cancer, or predictive of all-cause mortality, therefore RES did not substantially influenced health status and mortality risk of this population.	None reported
Thushara *et al*. [130]	2014	India	No study on RES effects: study on ameliorative efficacy of crocin on sesamol-induced platelet apoptosis			
Soare *et al.* [135]	2014	USA	6-month randomized, single-blind controlled trial, in 56 non-obese men and women, aged 38 to 55 years, assigned to a dietary supplement (SUP) group or control (CON) group (24 weeks of daily supplementation), with a 6-month follow-up	SUP group : 10 dietary supplements each day (100 mg RES, a complex of 800 mg each of green, black, and white tea extract, 250 mg of pomegranate extract, 650 mg of quercetin, 500 mg of acetyl-l-carnitine, 600 mg of lipoic acid, 900 mg of curcumin, 1 g of sesamin, 1.7 g of cinnamon bark extract, and 1 g fish oil).	No effect on arterial stiffness, endothelial function or blood pressure in nonobese men and women. No effect on key metabolic variables involved in aging and in CVD, including plasma markers of inflammation, oxidative stress and glycation, plasma lipids, growth factors, or body composition. Lack of beneficial metabolic effects perhaps related to the low phytochemical bioavailability or inadequate supplement potency of the phytochemicals.	Adverse events limited to mild gastro intestinal discomfort associated with taking the large number of oral supplements in 19% of the participants.
Micans [152]	2013	UK	Measurement of aortic wave velocity as a noninvasive method to evaluate the stiffness of arteries. Small clinical trial (20 patients) to appreciate the effects of supplements that appear to improve arterial stiffness	4 groups of patients: placebo, arginine, *trans*-RES (45 mg), l-carnosine, aminoguanidine: aortic wave pressure test before the supplement (baseline), and 1 h, 1 week and 1 month after supplement	Results for RES were given after 45 mg *trans*-RES given dialy for 5 weeks, showing an improvement of 15% in the aortic wave velocity test. No effect observed in the placebo roup.	None reported
Tomé-Carneiro *et al.* [125]	2013	Spain	Study of the molecular changes in peripheral blood mononuclear cells (PBMCs) associated to the one-year daily intake of a RES-enriched grape extract (GE-RES) in hypertensive male patients with type 2 diabetes mellitus, constituting a subset of 35 patients from a larger intervention study previously described [83]	Patients randomly allocated as follows: placebo (*n* = 9), GE (*n* = 13) and GE-RES (containing 8 mg RES) (*n* = 13)	Supplementation with GE or GE-RES did not affect body weight, blood pressure, glucose, HbA1c or lipids; no significant change in serum inflammatory markers, only a significant reduction of alkaline phosphatase and IL-6 levels. The expression of the pro-inflammatory cytokines CCL3, IL-1β and TNF-α was significantly reduced and that of the transcriptional repressor LRRFIP-1 increased in PBMCs from patients taking the GE-RES extract. A group of miRNAs involved in the regulation of the inflammatory response were found to be highly correlated and altered in the group consuming the GE-RES for 12 months.	None reported
Bo *et al.* [44]	2013	Italy	Randomized, double-blind, cross-over trial performed in 50 healthy adult smokers	25 subjects randomly allocated to “RES-first” (30-days: 500 mg RES/day, 30-days wash-out, 30-days placebo) and 25 to “placebo-first” (30-days placebo, 30-days wash-out, 30-days 500 mg RES/day)	RES significantly reduced C-reactive protein and triglyceride concentrations, and increased total antioxidant status values. No change in uric acid, glucose, insulin, cholesterol, liver enzyme concentrations, weight, waist circumference, and blood pressure.	None reported
Tomé-Carneiro *et al.* [92]	2013	Spain	Triple-blind, randomized, placebo-controlled, one-year follow-up, 3-arm pilot clinical trial of dose-depending effects of a RES-containing grape supplement on 75 stable patients with coronary artery disease treated according to guidelines for secondary prevention of CVD	3 arms: 350 mg/day of placebo, RES-containing grape extract (grape phenolics plus 8 mg RES) or conventional grape extract lacking RES during 6 months, and a double dose for the following 6 months	After 1 year, in contrast to the placebo and conventional grape extract groups, the RES-containing grape extract group showed an increase of the anti-inflammatory serum adiponectin and a decrease of the thrombogenic PAI-1; inhibition of atherothrombotic signals in peripheral blood mononuclear cells.	None reported
Popat *et al*. [153]	2013	UK	Not applicable: phase 2 study of SRT501 (RES) with bortezomib for patients with relapsed and or refractory multiple myeloma			
Militaru *et al.* [95]	2013	Romania	Randomized, double-blinded, active-controlled, parallel clinical trial with 60 days oral supplementation with calcium fructoborate, RES, and their combination, in 166 subjects with stable angina pectoris	3 groups (with usual medical care and treatment): group 1 received a single daily capsule with RES 20 mg/day (10 mg trans-RES); group 2 received a single daily capsule of RES 20 mg/day (10 mg trans-RES) combined with calcium fructoborate (CF) 112 mg/day (3 mg boron); group 3 received a single daily capsule of CF 112 mg/day (3 mg boron). Non-randomized control group: only usual medical care and treatment.	Significant decrease of hs-CRP in all groups at the 30-day and 60-day visits, greater for group 3, followed by group 2. The NT-proBNP was significantly lowered in groups 1 and 3, but combination RES + CF (group 2) was the most effective. Lipid markers showed slight changes from baseline in all groups. Improvement in the quality of life: best for group 2.	None reported
Agarwal *et al.* [154]	2013	USA	Double-blind, randomized, placebo-controlledtudy of the effects of RES on endothelial response and plasma biomarkers in 44 healthy individuals using a novel unbiased assay to assess the overall inflammatory capacity of plasma on expression of genes associated with inflammation and atherosclerosis	1 month treatment RES supplement : 400 mg trans-RES (98% pure, from Polygonum Cuspidatum), 400 mg of mixed Bordeaux whole grape extract (90% polyphenols), and 100 mg quercetin	Exposing cultured human coronary artery endothelial cells to plasma drawn post-RES resulted in significantly lower mRNA expression of VCAM, ICAM, and IL-8 than plasma drawn from the same subjects at baseline (no effect of placebo). Significant reduction in plasma interferon-γ with RES supplement (not with placebo), and significant reduction in fasting insulin concentration with RES supplement	2 subjects with RES and 1 with placebo reported mild gastrointestinal side effects
Tomé-Carneiro *et al.* [45]	2012	Spain	Study of the effect of a grape supplement in oxidized LDL (LDLox), apolipoprotein-B (ApoB), and serum lipids on 75 statin-treated patients in primary CVD prevention	Grape exctract containing 8 mg RES. 3 parallel arms: one capsule (350 mg) daily for 6 months containing RES-enriched grape extract (GE-RES, Stilvid^®^), grape extract (GE, similar polyphenolic content but no RES), or placebo (maltodextrin)	LDL-cholesterol, ApoB, LDLox and LDLox/ApoB decreased in the Stilvid^®^ group, whereas the ratio non-HDL-cholesterol/ApoB increased, which might exert additional cardioprotection	None reported
Tomé-Carneiro *et al.* [155]	2012	Spain	75 patients undergoing primary prevention of CVD participated in this triple-blinded, randomized, parallel, dose-response, placebo-controlled, 1-year follow-up trial: effects of a dietary RES-rich grape supplement on the inflammatory and fibrinolytic status	3 groups: placebo (maltodextrin), or RES-rich grape supplement (8 mg RES), or conventional grape supplement lacking RES, for the first 6 months, and a double dose for the next 6 months	1-year consumption of a RES-rich grape supplement improved the inflammatory and fibrinolytic status in patients who were on statins for primary prevention of CVD and at high CVD risk (*i.e.*, with diabetes or hypercholesterolemia plus ≥1 other CV risk factor)	None reported
Zamora-Ros *et al.* [156]	2012	Spain	Study of the associations between total urinary RES metabolites (TRMs) as biomarkers of wine and RES consumption and CV risk factors in a large cross-sectional study including high CV risk individuals (1000 participants—479 men and 521 women—of the PREDIMED Study)	TRMs were used as a biomarker of wine consumption (when the model was not adjusted for alcohol intake) or as biomarkers of RES intake (when the model was additionally adjusted for alcohol intake)	Both RES and wine intake, evaluated as TRMs, were associated with beneficial changes in blood lipid profiles, fasting blood glucose (only RES) and heart rate, suggesting that RES intake via wine consumption might help to decrease CV risk factors	None reported
Magyar *et al.* [93]	2012	Hungary	Double-blind, placebo controlled trial conducted in 40 post-infarction Caucasian patients to investigate if RES had a clinically measurable cardioprotective effect	10 mg RES daily (capsule) for 3 months	RES improved left ventricle diastolic function, endothelial function, lowered LDL-cholesterol level and protected against unfavourable hemorheological changes measured in patients with coronary artery disease	None reported
Fujitaka *et al.* [157]	2011	Japan	Study of Longevinex, a modified RES, on endothelial function in 34 patients with metabolic syndrome	2 groups: group A, with Longevinex administered for 3 months and then discontinued for 3 months, and group B, with Longevinex administered between 3 and 6 months. 1 capsule of Longevinex daily, containing 100 mg trans-RES	Longevinex specifically improved endothelial function (flow-mediated dilatation) in subjects with metabolic syndrome	None reported
Wong *et al.* [158]	2011	Australia	Is consumption of RES able to improve flow-mediated dilatation (FMD) of the brachial artery is a biomarker of endothelial function, in 19 overweight/obese (BMI 25–35) men or post-menopausal women, aged 30–70 years, with untreated borderline hypertension (systolic BP: 130–160 mmHg or diastolic BP: 85–100 mmHg)	3 doses of RES in RES capsules (resVida™ 30, 90 and 270 mg) or placebo capsules, in randomised, double-blind, placebo-controlled, crossover human intervention trial comprising 4 visits at weekly intervals (note that the lowest RES dose (30 mg) used in this study cannot be obtained from normal dietary habits)	First study to evaluate the acute effects of RES consumption on human circulatory function: significant dose effect of RES on plasma RES concentration (*p* < 0.001) and on FMD (*p* < 0.01), which increased from 4.1 ± 0.8% (placebo) to 7.7 ± 1.5% after 270 mg RES	None reported
Karlsen *et al.* [159]	2010	Norway	Effect of bilberry juice (RES content: 1–12 mg/100 g fw) on serum and plasma biomarkers of inflammation and antioxidant status in men aged between 30 and 70 years and women between 45 and 70 years and at least 12 months postmenopausal, at elevated risk of CVD	One group consumed 330 mL bilberry juice/day (diluted in 1 L water) and one group consumed 1 L water/day	Supplementation with bilberry juice resulted in significant decreases in plasma concentrations of C-reactive protein (CRP), interleukin (IL)-6, IL-15, and monokine induced by interferon-g (MIG)	None reported
Gresele *et al.* [160]	2008	Italy	Study of the effects of RES, at concentrations attainable after moderate wine intake, on platelet ^●^NO production in 20 healthy volunteers. Moreover, RES at the concentrations detected in plasma after wine intake, was incubated in vitro with washed platelets and several variables related to ^●^NO production and to signal transduction were measured	Study before and after 15 days of controlled white or red wine intake (300 mL/day): total polyphenolic concentration = 1.8 g/L for red wine and 0.25 g/L for white wine. Maximum RES concentration for in vitro studies: 0.5 μmol/L	After wine intake, increase of plasma RES and release of ^●^NO by stimulated platelets. In vitro, RES enhanced production of ^●^NO by stimulated platelets, activity of platelet ^●^NO synthase (NOS), phosphorylation of protein kinase B, an activator of the endothelial NOS (eNOS), and phosphorylation of vasodilator-activated protein.	None reported
Zamora-Ros *et al.* [131]	2006	Spain	Analysis of urinary total RES metabolites (TRMs) as a biomarker of moderate wine (sparkling, white, or red wine) consumption, in 2 open, prospective, randomized, crossover, single-blinded clinical trials	Daily amount of total RES (for 4 weeks): 0.357, 0.398, and 2.56 mg for sparkling, white, and red wine, respectively	RES metabolites in urine may be useful biomarkers of wine intake in epidemiologic and intervention studies (the aim of the study was not to report beneficial effect of RES in patients with CV risk)	None reported
Cruz *et al.* [127]	2006	Sweden	Investigation of acute vasodilator responses to phytoestrogens and selective estrogen receptor-alpha (ERalpha) agonist in isolated small arteries from 15 men (38–71 years) with established coronary heart disease (CHD) and with a history of MI (1–12 months before study) *vs.* healthy male control subjects	10-30 µM RES	Phytoestrogens (especially RES), at concentrations achievable by ingestion of phytoestrogen-rich food products, induced dilatation *ex vivo* of small peripheral arteries from normal men and from those with established CHD. The contribution of ^●^NO to dilatory responses by these compounds is pertinent to arteries from control males, whereas other ^●^NO-independent dilatory mechanism(s) are involved in arteries from CHD	Not applicable (*ex vivo* study)
Lekakis *et al.* [161]	2005	Greece	30 male patients with coronary heart disease, randomly assigned either to a red grape polyphenol extract dissolved in 20 mL of water (*n* = 15) or 20 mL of water (placebo) (*n* = 15), to examine whether acute intake of the extract has a positive effect on brachial artery flow-mediated dilatation	Use of 600 mg extract containing 0.9 mg trans-RES and other polyphenolic compounds	Intake of the extract caused an increase in flow-mediated dilatation, which was significantly higher than the baseline values. No change was observed after intake of placebo.	None reported (the long-term effect of the extract on endothe-lial function has not been studied)
Rakici *et al.* [128]	2005	Turkey	First study of the relaxant effect of RES on human blood vessels of internal mammary artery and saphenous vein grafts from 38 randomized male patients undergoing coronary artery revascularization	10–70 μM RES	70 µM RES caused relaxation in saphenous vein and internal mammary artery (mainly endothelium-dependent and ^●^NO-mediated relaxations in internal mammary artery, partially in saphenous vein rings)	Not applica-ble (*ex vivo* study)

## References

[1] Laslett L.J., Alagona P., Clark B.A., Drozda J.P., Saldivar F., Wilson S.R., Poe C., Hart M. (2012). The worldwide environment of cardiovascular disease: Prevalence, diagnosis, therapy, and policy issues: A report from the American College of Cardiology. J. Am. Coll. Cardiol..

[2] Renaud S., de Lorgeril M. (1992). Wine, alcohol, platelets, and the French paradox for coronary heart disease. Lancet.

[3] Smoliga J.M., Baur J.A., Hausenblas H.A. (2011). Resveratrol and health—A comprehensive review of human clinical trials. Mol. Nutr. Food Res..

[4] Delmas D., Jannin B., Latruffe N. (2005). Resveratrol: Preventing properties against vascular alterations and ageing. Mol. Nutr. Food Res..

[5] Zordoky B.N., Robertson I.M., Dyck J.R. (2015). Preclinical and clinical evidence for the role of resveratrol in the treatment of cardiovascular diseases. Biochim. Biophys. Acta.

[6] Cottart C.H., Nivet-Antoine V., Beaudeux J.L. (2014). Review of recent data on the metabolism, biological effects, and toxicity of resveratrol in humans. Mol. Nutr. Food Res..

[7] Wallerath T., Deckert G., Ternes T., Anderson H., Li H., Witte K., Förstermann U. (2002). Resveratrol, a polyphenolic phytoalexin present in red wine, enhances expression and activity of endothelial nitric oxide synthase. Circulation.

[8] Leikert J.F., Räthel T.R., Wohlfart P., Cheynier V., Vollmar A.M., Dirsch V.M. (2002). Red wine polyphenols enhance endothelial nitric oxide synthase expression and subsequent nitric oxide release from endothelial cells. Circulation.

[9] Saad M.I., Abdelkhalek T.M., Saleh M.M., Kamel M.A., Youssef M., Tawfik S.H., Dominguez H. (2015). Insights into the molecular mechanisms of diabetes-induced endothelial dysfunction: Focus on oxidative stress and endothelial progenitor cells. Endocrine.

[10] Lin K.Y., Ito A., Asagami T., Tsao P.S., Adimoolam S., Kimoto M., Tsuji H., Reaven G.M., Cooke J.P. (2002). Impaired nitric oxide synthase pathway in diabetes mellitus: Role of asymmetric dimethylarginine and dimethylarginine dimethylaminohydrolase. Circulation.

[11] Li H., Förstermann U. (2000). Nitric oxide in the pathogenesis of vascular disease. J. Pathol..

[12] Camont L., Collin F., Marchetti C., Jore D., Gardes-Albert M., Bonnefont-Rousselot D. (2010). Liquid chromatographic/electrospray ionization mass spectrometric identification of the oxidation end-products of trans-resveratrol in aqueous solutions. Rapid Commun. Mass Spectrom..

[13] Camont L., Collin F., Couturier M., Thérond P., Jore D., Gardès-Albert M., Bonnefont-Rousselot D. (2012). Radical-induced oxidation of trans-resveratrol. Biochimie.

[14] Rhayem Y., Thérond P., Camont L., Couturier M., Beaudeux J.L., Legrand A., Jore D., Gardés-Albert M., Bonnefont-Rousselot D. (2008). Chain-breaking activity of resveratrol and piceatannol in a linoleate micellar model. Chem. Phys. Lipids.

[15] Scalera F., Kielstein J.T., Martens-Lobenhoffer J., Postel S.C., Täger M., Bode-Böger S.M. (2005). Erythropoietin increases asymmetric dimethylarginine in endothelial cells: Role of dimethylarginine dimethylaminohydrolase. J. Am. Soc. Nephrol..

[16] Frombaum M., le Clanche S., Bonnefont-Rousselot D., Borderie D. (2012). Antioxidant effects of resveratrol and other stilbene derivatives on oxidative stress and ^•^NO bioavailability: Potential benefits to cardiovascular diseases. Biochimie.

[17] Frombaum M., Thérond P., Djelidi R., Beaudeux J.L., Bonnefont-Rousselot D., Borderie D. (2011). Piceatannol is more effective than resveratrol in restoring endothelial cell dimethylargininedimethylaminohydrolase expression and activity after high-glucose oxidative stress. Free Radic. Res..

[18] Bivalacqua T.J., Hellstrom W.J., Kadowitz P.J., Champion H.C. (2001). Increased expression of arginase II in human diabetic corpus cavernosum: In diabetic-associated erectile dysfunction. Biochem. Biophys. Res. Commun..

[19] White A.R., Ryoo S., Li D., Champion H.C., Steppan J., Wang D., Nyhan D., Shoukas A.A., Hare J.M., Berkowitz D.E. (2006). Knockdown of arginase I restores NO signaling in the vasculature of old rats. Hypertension.

[20] Hong L., Fast W. (2007). Inhibition of human dimethylarginine dimethylaminohydrolase-1 by *S*-nitroso-l-homocysteine and hydrogen peroxide. Analysis, quantification, and implications for hyperhomocysteinemia. J. Biol. Chem..

[21] Yuan Q., Peng J., Liu S.Y., Wang C.J., Xiang D.X., Xiong X.M., Hu C.P., Li Y.J. (2010). Inhibitory effect of resveratrol derivative BTM-0512 on high glucose-induced cell senescence involves dimethylaminohydrolase/asymmetric dimethylarginine pathway. Clin. Exp. Pharmacol. Physiol..

[22] Dolinsky V.W., Dyck J.R. (2011). Calorie restriction and resveratrol in cardiovascular health and disease. Biochim. Biophys. Acta.

[23] Li H., Xia N., Förstermann U. (2012). Cardiovascular effects and molecular targets of resveratrol. Nitric Oxide.

[24] Fichtlscherer S., Zeiher A.M., Dimmeler S. (2011). Circulating microRNAs: Biomarkers or mediators of cardiovascular diseases?. Arterioscler. Thromb. Vasc. Biol..

[25] Mukhopadhyay P., Pacher P., Das D.K. (2011). MicroRNA signatures of resveratrol in the ischemic heart. Ann. N. Y. Acad. Sci..

[26] Tomé-Carneiro J., Larrosa M., González-Sarrías A., Tomás-Barberán F.A., García-Conesa M.T., Espín J.C. (2013). Resveratrol and clinical trials: The crossroad from *in vitro* studies to human evidence. Curr. Pharm. Des..

[27] Glass C.K., Witztum J.L. (2001). Atherosclerosis. The road ahead. Cell.

[28] Göçmen A.Y., Burgucu D., Gümüşlü S. (2011). Effect of resveratrol on platelet activation in hypercholesterolemic rats: CD40-CD40L system as a potential target. Appl. Physiol. Nutr. Metab..

[29] Cho I.J., Ahn J.Y., Kim S., Choi M.S., Ha T.Y. (2008). Resveratrol attenuates the expression of HMG-CoA reductase mRNA in hamsters. Biochem. Biophys. Res. Commun..

[30] Yashiro T., Nanmoku M., Shimizu M., Inoue J., Sato R. (2012). Resveratrol increases the expression and activity of the low density lipoprotein receptor in hepatocytes by the proteolytic activation of the sterol regulatory element-binding proteins. Atherosclerosis.

[31] Witztum J.L., Steinberg D. (1991). Role of oxidized low density lipoprotein in atherogenesis. J. Clin. Investig..

[32] Berrougui H., Grenier G., Loued S., Drouin G., Khalil A. (2009). A new insight into resveratrol as an atheroprotective compound: Inhibition of lipid peroxidation and enhancement of cholesterol efflux. Atherosclerosis.

[33] Ramprasath V.R., Jones P.J. (2010). Anti-atherogenic effects of resveratrol. Eur. J. Clin. Nutr..

[34] Lin Y.C., Chen L.H., Varadharajan T., Tsai M.J., Chia Y.C., Yuan T.C., Sung P.J., Weng C.F. (2014). Resveratrol inhibits glucose-induced migration of vascular smooth muscle cells mediated by focal adhesion kinase. Mol. Nutr. Food Res..

[35] Haskó G., Pacher P. (2010). Endothelial Nrf2 activation: A new target for resveratrol?. Am. J. Physiol. Heart Circ. Physiol..

[36] Deng Y.H., Alex D., Huang H.Q., Wang N., Yu N., Wang Y.T., Leung G.P., Lee S.M. (2011). Inhibition of TNF-α-mediated endothelial cell-monocyte cell adhesion and adhesion molecules expression by the resveratrol derivative, trans-3,5,4′-trimethoxystilbene. Phytother. Res..

[37] Park D.W., Baek K., Kim J.R., Lee J.J., Ryu S.H., Chin B.R., Baek S.H. (2009). Resveratrol inhibits foam cell formation via NADPH oxidase 1- mediated reactive oxygen species and monocyte chemotactic protein-1. Exp. Mol. Med..

[38] Latruffe N., Lançon A., Frazzi R., Aires V., Delmas D., Michaille J.J., Djouadi F., Bastin J., Cherkaoui-Malki M. (2015). Exploring new ways of regulation by resveratrol involving miRNAs, with emphasis on inflammation. Ann. N. Y. Acad. Sci..

[39] Hao E., Lang F., Chen Y., Zhang H., Cong X., Shen X., Su G. (2013). Resveratrol alleviates endotoxin-induced myocardial toxicity via the Nrf2 transcription factor. PLoS ONE.

[40] El-Mowafy A.M., Alkhalaf M., El-Kashef H.A. (2008). Resveratrol reverses hydrogen peroxide-induced proliferative effects in human coronary smooth muscle cells: A novel signaling mechanism. Arch. Med. Res..

[41] Sahebkar A. (2013). Effects of resveratrol supplementation on plasma lipids: A systematic review and meta-analysis of randomized controlled trials. Nutr. Rev..

[42] Bhatt J.K., Thomas S., Nanjan M.J. (2012). Resveratrol supplementation improves glycemic control in type 2 diabetes mellitus. Nutr. Res..

[43] Timmers S., Konings E., Bilet L., Houtkooper R.H., van de Weijer T., Goossens G.H., Hoeks J., van der Krieken S., Ryu D., Kersten S. (2011). Calorie restriction-like effects of 30 days of resveratrol supplementation on energy metabolism and metabolic profile in obese humans. Cell Metab..

[44] Bo S., Ciccone G., Castiglione A., Gambino R., de Michieli F., Villois P., Durazzo M., Cavallo-Perin P., Cassader M. (2013). Anti-inflammatory and antioxidant effects of resveratrol in healthy smokers a randomized, double-blind, placebo-controlled, cross-over trial. Curr. Med. Chem..

[45] Tomé-Carneiro J., Gonzálvez M., Larrosa M., García-Almagro F.J., Avilés-Plaza F., Parra S., Yáñez-Gascón M.J., Ruiz-Ros J.A., García-Conesa M.T., Tomás-Barberán F.A. (2012). Consumption of a grape extract supplement containing resveratrol decreases oxidized LDL and ApoB in patients undergoing primary prevention of cardiovascular disease: A triple-blind, 6-month follow-up, placebo-controlled, randomized trial. Mol. Nutr. Food Res..

[46] Smulyan H., Mookherjee S., Safar M.E. (2016). The two faces of hypertension: Role of aortic stiffness. J. Am. Soc. Hypertens..

[47] Rivera L., Morón R., Zarzuelo A., Galisteo M. (2009). Long-term resveratrol administration reduces metabolic disturbances and lowers blood pressure in obese Zucker rats. Biochem. Pharmacol..

[48] Dolinsky V.W., Chakrabarti S., Pereira T.J., Oka T., Levasseur J., Beker D., Zordoky B.N., Morton J.S., Nagendran J., Lopaschuk G.D. (2013). Resveratrol prevents hypertension and cardiac hypertrophy in hypertensive rats and mice. Biochim. Biophys. Acta.

[49] Chan V., Fenning A., Iyer A., Hoey A., Brown L. (2011). Resveratrol improves cardiovascular function in DOCA-salt hypertensive rats. Curr. Pharm. Biotechnol..

[50] Rimbaud S., Ruiz M., Piquereau J., Mateo P., Fortin D., Veksler V., Garnier A., Ventura-Clapier R. (2011). Resveratrol improves survival, hemodynamics and energetics in a rat model of hypertension leading to heart failure. PLoS ONE.

[51] Thandapilly S.J., Louis X.L., Behbahani J., Movahed A., Yu L., Fandrich R., Zhang S., Kardami E., Anderson H.D., Netticadan T. (2013). Reduced hemodynamic load aids low-dose resveratrol in reversing cardiovascular defects in hypertensive rats. Hypertens. Res..

[52] Gordish K.L., Beierwaltes W.H. (2014). Resveratrol induces acute endothelium-dependent renal vasodilation mediated through nitric oxide and reactive oxygen species scavenging. Am. J. Physiol. Ren. Physiol..

[53] Arunachalam G., Yao H., Sundar I.K., Caito S., Rahman I. (2010). SIRT1 regulates oxidant- and cigarette smoke-induced eNOS acetylation in endothelial cells: Role of resveratrol. Biochem. Biophys. Res. Commun..

[54] Soylemez S., Sepici A., Akar F. (2009). Resveratrol supplementation gender independently improves endothelial reactivity and suppresses superoxide production in healthy rats. Cardiovasc. Drugs Ther..

[55] Rush J.W., Quadrilatero J., Levy A.S., Ford R.J. (2007). Chronic resveratrol enhances endothelium-dependent relaxation but does not alter eNOS levels in aorta of spontaneously hypertensive rats. Exp. Biol. Med. (Maywood).

[56] Cao X., Luo T., Luo X., Tang Z. (2014). Resveratrol prevents AngII-induced hypertension via AMPK activation and RhoA/ROCK suppression in mice. Hypertens. Res..

[57] Liu Y., Ma W., Zhang P., He S., Huang D. (2015). Effect of resveratrol on blood pressure: A meta-analysis of randomized controlled trials. Clin. Nutr..

[58] Carrizzo A., Puca A., Damato A., Marino M., Franco E., Pompeo F., Traficante A., Civitillo F., Santini L., Trimarco V. (2013). Resveratrol improves vascular function in patients with hypertension and dyslipidemia by modulating NO metabolism. Hypertension.

[59] Clark D., Tuor U.I., Thompson R., Institoris A., Kulynych A., Zhang X., Kinniburgh D.W., Bari F., Busija D.W., Barber P.A. (2012). Protection against recurrent stroke with resveratrol: Endothelial protection. PLoS ONE.

[60] Huang S.S., Tsai M.C., Chih C.L., Hung L.M., Tsai S.K. (2001). Resveratrol reduction of infarct size in Long-Evans rats subjected to focal cerebral ischemia. Life Sci..

[61] Arrick D.M., Sun H., Patel K.P., Mayhan W.G. (2011). Chronic resveratrol treatment restores vascular responsiveness of cerebral arterioles in type 1 diabetic rats. Am. J. Physiol. Heart Circ. Physiol..

[62] Singh N., Agrawal M., Doré S. (2013). Neuroprotective properties and mechanisms of resveratrol in *in vitro* and *in vivo* experimental cerebral stroke models. ACS Chem. Neurosci..

[63] Wan D., Zhou Y., Wang K., Hou Y., Hou R., Ye X. (2016). Resveratrol provides neuroprotection by inhibiting phosphodiesterases and regulating the cAMP/AMPK/SIRT1 pathway after stroke in rats. Brain Res. Bull..

[64] Kennedy D.O., Wightman E.L., Reay J.L., Lietz G., Okello E.J., Wilde A., Haskell C.F. (2010). Effects of resveratrol on cerebral blood flow variables and cognitive performance in humans: A double-blind, placebo-controlled, crossover investigation. Am. J. Clin. Nutr..

[65] Wightman E.L., Reay J.L., Haskell C.F., Williamson G., Dew T.P., Kennedy D.O. (2014). Effects of resveratrol alone or in combination with piperine on cerebral blood flow parameters and cognitive performance in human subjects: A randomised, double-blind, placebo-controlled, cross-over investigation. Br. J. Nutr..

[66] Evans H.M., Howe P.R., Wong R.H. (2016). Clinical evaluation of effects of chronic resveratrol supplementation on cerebrovascular function, cognition, mood, physical function and general well-being in postmenopausal women-rationale and study design. Nutrients.

[67] Raj P., Louis X.L., Thandapilly S.J., Movahed A., Zieroth S., Netticadan T. (2014). Potential of resveratrol in the treatment of heart failure. Life Sci..

[68] Hung L.M., Su M.J., Chen J.K. (2004). Resveratrol protects myocardial ischemia-reperfusion injury through both NO-dependent and NO-independent mechanisms. Free Radic. Biol. Med..

[69] Shen M., Jia G.L., Wang Y.M., Ma H. (2006). Cardioprotective effect of resvaratrol pretreatment on myocardial ischemia-reperfusion induced injury in rats. Vasc. Pharmacol..

[70] Shalwala M., Zhu S.G., Das A., Salloum F.N., Xi L., Kukreja R.C. (2014). Sirtuin 1 (SIRT1) activation mediates sildenafil induced delayed cardioprotection against ischemia-reperfusion injury in mice. PLoS ONE.

[71] Das S., Cordis G.A., Maulik N., Das D.K. (2005). Pharmacological preconditioning with resveratrol: Role of CREB-dependent Bcl-2 signaling via adenosine A3 receptor activation. Am. J. Physiol. Heart Circ. Physiol..

[72] Kaga S., Zhan L., Matsumoto M., Maulik N. (2005). Resveratrol enhances neovascularization in the infarcted rat myocardium through the induction of thioredoxin-1, heme oxygenase-1 and vascular endothelial growth factor. J. Mol. Cell. Cardiol..

[73] Fukuda S., Kaga S., Zhan L., Bagchi D., Das D.K., Bertelli A., Maulik N. (2006). Resveratrol ameliorates myocardial damage by inducing vascular endothelial growth factor-angiogenesis and tyrosine kinase receptor Flk-1. Cell Biochem. Biophys..

[74] Chen Y.R., Yi F.F., Li X.Y., Wang C.Y., Chen L., Yang X.C., Su P.X., Cai J. (2008). Resveratrol attenuates ventricular arrhythmias and improves the long-term survival in rats with myocardial infarction. Cardiovasc. Drugs Ther..

[75] Robich M.P., Osipov R.M., Chu L.M., Han Y., Feng J., Nezafat R., Clements R.T., Manning W.J., Sellke F.W. (2011). Resveratrol modifies risk factors for coronary artery disease in swine with metabolic syndrome and myocardial ischemia. Eur. J. Pharmacol..

[76] Gurusamy N., Lekli I., Mukherjee S., Ray D., Ahsan M.K., Gherghiceanu M., Popescu L.M., Das D.K. (2010). Cardioprotection by resveratrol: A novel mechanism via autophagy involving the mTORC2 pathway. Cardiovasc. Res..

[77] Xuan W., Wu B., Chen C., Chen B., Zhang W., Xu D., Bin J., Liao Y. (2012). Resveratrol improves myocardial ischemia and ischemic heart failure in mice by antagonizing the detrimental effects of fractalkine. Crit. Care Med..

[78] Gu X.S., Wang Z.B., Ye Z., Lei J.P., Li L., Su D.F., Zheng X. (2014). Resveratrol, an activator of SIRT1, upregulates AMPK and improves cardiac function in heart failure. Genet. Mol. Res..

[79] Kanamori H., Takemura G., Goto K., Tsujimoto A., Ogino A., Takeyama T., Kawaguchi T., Watanabe T., Morishita K., Kawasaki M. (2013). Resveratrol reverses remodeling in hearts with large, old myocardial infarctions through enhanced autophagy-activating AMP kinase pathway. Am. J. Pathol..

[80] Sabe A.A., Elmadhun N.Y., Dalal R.S., Robich M.P., Sellke F.W. (2014). Resveratrol regulates autophagy signaling in chronically ischemic myocardium. J. Thorac. Cardiovasc. Surg..

[81] Shen M., Wu R.X., Zhao L., Li J., Guo H.T., Fan R., Cui Y., Wang Y.M., Yue S.Q., Pei J.M. (2012). Resveratrol attenuates ischemia/reperfusion injury in neonatal cardiomyocytes and its underlying mechanism. PLoS ONE.

[82] Sabe A.A., Sadek A.A., Elmadhun N.Y., Dalal R.S., Robich M.P., Bianchi C., Sellke F.W. (2015). Investigating the effects of resveratrol on chronically ischemic myocardium in a swine model of metabolic syndrome: A proteomics analysis. J. Med. Food.

[83] Dong W., Yang R., Yang J., Yang J., Ding J., Wu H., Zhang J. (2015). Resveratrol pretreatment protects rat hearts from ischemia/reperfusion injury partly via a NALP3 inflammasome pathway. Int. J. Clin. Exp. Pathol..

[84] Mukhopadhyay P., Mukherjee S., Ahsan K., Bagchi A., Pacher P., Das D.K. (2010). Restoration of altered microRNA expression in the ischemic heart with resveratrol. PLoS ONE.

[85] Thum T., Gross C., Fiedler J., Fischer T., Kissler S., Bussen M., Galuppo P., Just S., Rottbauer W., Frantz S. (2008). MicroRNA-21 contributes to myocardial disease by stimulating MAP kinase signaling in fibroblasts. Nature.

[86] Roy S., Khanna S., Hussain S.R., Biswas S., Azad A., Rink C., Gnyawali S., Shilo S., Nuovo G.J., Sen C.K. (2009). MicroRNA expression in response to murine myocardial infarction: miR-21 regulates fibroblast metalloprotease-2 via phosphatase and tensin homologue. Cardiovasc. Res..

[87] Cascio S., D’Andrea A., Ferla R., Surmacz E., Gulotta E., Amodeo V., Bazan V., Gebbia N., Russo A. (2010). miR-20b modulates VEGF expression by targeting HIF-1 alpha and STAT3 in MCF-7 breast cancer cells. J. Cell. Physiol..

[88] Guttilla I.K., White B.A. (2009). Coordinate regulation of FOXO1 by miR-27a, miR-96, and miR-182 in breast cancer cells. J. Biol. Chem..

[89] Saunders L.R., Sharma A.D., Tawney J., Nakagawa M., Okita K., Yamanaka S., Willenbring H., Verdin E. (2010). miRNAs regulate SIRT1 expression during mouse embryonic stem cell differentiation and in adult mouse tissues. Aging (Albany NY).

[90] Van Rooij E., Sutherland L.B., Thatcher J.E., DiMaio J.M., Naseem R.H., Marshall W.S., Hill J.A., Olson E.N. (2008). Dysregulation of microRNAs after myocardial infarction reveals a role of miR-29 in cardiac fibrosis. Proc. Natl. Acad. Sci. USA.

[91] Yin K., Zhao L., Feng D., Ma W., Liu Y., Wang Y., Liang J., Yang F., Bi C., Chen H. (2015). Resveratrol attenuated low ambient temperature-induced myocardial hypertrophy via inhibiting cardiomyocyte apoptosis. Cell. Physiol. Biochem..

[92] Tomé-Carneiro J., Gonzálvez M., Larrosa M., Yáñez-Gascón M.J., García-Almagro F.J., Ruiz-Ros J.A., Tomás-Barberán F.A., García-Conesa M.T., Espín J.C. (2013). Grape resveratrol increases serum adiponectin and downregulates inflammatory genes in peripheral blood mononuclear cells: A triple-blind, placebo-controlled, one-year clinical trial in patients with stable coronary artery disease. Cardiovasc. Drugs Ther..

[93] Magyar K., Halmosi R., Palfi A., Feher G., Czopf L., Fulop A., Battyany I., Sumegi B., Toth K., Szabados E. (2012). Cardioprotection by resveratrol: A human clinical trial in patients with stable coronary artery disease. Clin. Hemorheol. Microcirc..

[94] Walle T., Hsieh F., DeLegge M.H., Oatis J.E., Walle U.K. (2004). High absorption but very low bioavailability of oral resveratrol in humans. Drug Metab. Dispos..

[95] Militaru C., Donoiu I., Craciun A., Scorei I.D., Bulearca A.M., Scorei R.I. (2013). Oral resveratrol and calcium fructoborate supplementation in subjects with stable angina pectoris: Effects on lipid profiles, inflammation markers, and quality of life. Nutrition.

[96] Gupta P.K., DiPette D.J., Supowit S.C. (2014). Protective effect of resveratrol against pressure overload-induced heart failure. Food Sci. Nutr..

[97] Tanno M., Kuno A., Yano T., Miura T., Hisahara S., Ishikawa S., Shimamoto K., Horio Y. (2010). Induction of manganese superoxide dismutase by nuclear translocation and activation of SIRT1 promotes cell survival in chronic heart failure. J. Biol. Chem..

[98] Chan A.Y., Dolinsky V.W., Soltys C.L., Viollet B., Baksh S., Light P.E., Dyck J.R. (2008). Resveratrol inhibits cardiac hypertrophy via AMP-activated protein kinase and Akt. J. Biol. Chem..

[99] Sulaiman M., Matta M.J., Sunderesan N.R., Gupta M.P., Periasamy M., Gupta M. (2010). Resveratrol, an activator of SIRT1, upregulates sarcoplasmic calcium ATPase and improves cardiac function in diabetic cardiomyopathy. Am. J. Physiol. Heart Circ. Physiol..

[100] Dolinsky V.W., Soltys C.L., Rogan K.J., Chan A.Y., Nagendran J., Wang S., Dyck J.R. (2015). Resveratrol prevents pathological but not physiological cardiac hypertrophy. J. Mol. Med. (Berl.).

[101] Azorín-Ortuño M., Yáñez-Gascón M.J., Vallejo F., Pallarés F.J., Larrosa M., Lucas R., Morales J.C., Tomás-Barberán F.A., García-Conesa M.T., Espín J.C. (2011). Metabolites and tissue distribution of resveratrol in the pig. Mol. Nutr. Food Res..

[102] Bresciani L., Calani L., Bocchi L., Delucchi F., Savi M., Ray S., Brighenti F., Stilli D., del Rio D. (2014). Bioaccumulation of resveratrol metabolites in myocardial tissue is dose-time dependent and related to cardiac hemodynamics in diabetic rats. Nutr. Metab. Cardiovasc. Dis..

[103] Brantley S.J., Argikar A.A., Lin Y.S., Nagar S., Paine M.F. (2014). Herb-drug interactions: Challenges and opportunities for improved predictions. Drug Metab. Dispos..

[104] Cottart C.H., Nivet-Antoine V., Laguillier-Morizot C., Beaudeux J.L. (2010). Resveratrol bioavailability and toxicity in humans. Mol. Nutr. Food Res..

[105] Goldberg D.M., Yan J., Soleas G.J. (2003). Absorption of three wine-related polyphenols in three different matrices by healthy subjects. Clin. Biochem..

[106] Calamini B., Ratia K., Malkowski M.G., Cuendet M., Pezzuto J.M., Santarsiero B.D., Mesecar A.D. (2010). Pleiotropic mechanisms facilitated by resveratrol and its metabolites. Biochem. J..

[107] Boocock D.J., Faust G.E., Patel K.R., Schinas A.M., Brown V.A., Ducharme M.P., Booth T.D., Crowell J.A., Perloff M., Gescher A.J. (2007). Phase I dose escalation pharmacokinetic study in healthy volunteers of resveratrol, a potential cancer chemopreventive agent. Cancer Epidemiol. Biomark. Prev..

[108] Das S., Ng K.Y. (2010). Impact of glutaraldehyde on *in vivo* colon-specific release of resveratrol from biodegradable pectin-based formulation. J. Pharm. Sci..

[109] Lu X., Ji C., Xu H., Li X., Ding H., Ye M., Zhu Z., Ding D., Jiang X., Ding X. (2009). Resveratrol-loaded polymeric micelles protect cells from Abeta-induced oxidative stress. Int. J. Pharm..

[110] Amri A., le Clanche S., Thérond P., Bonnefont-Rousselot D., Borderie D., Lai-Kuen R., Chaumeil J.C., Sfar S., Charrueau C. (2014). Resveratrol self-emulsifying system increases the uptake by endothelial cells and improves protection against oxidative stress-mediated death. Eur. J. Pharm. Biopharm..

[111] Neves A.R., Martins S., Segundo M.A., Reis S. (2016). Nanoscale delivery of resveratrol towards enhancement of supplements and nutraceuticals. Nutrients.

[112] Diaz-Gerevini G.T., Repossi G., Dain A., Tarres M.C., Das U.N., Eynard A.R. (2016). Beneficial action of resveratrol: How and why?. Nutrition.

[113] Penalva R., Esparza I., Larraneta E., González-Navarro C.J., Gamazo C., Irache J.M. (2015). Zein-based nanoparticles improve the oral bioavailability of resveratrol and its anti-inflammatory effects in a mouse model of endotoxic shock. J. Agric. Food Chem..

[114] Da Rocha Lindner G., Santos D.B., Colle D., Moreira E.L.G., Prediger R.D., Farina M., Khalil N.M., Mainardes R.M. (2015). Improved neuroprotective effects of resveratrol-loadedpolysorbate 80-coated poly (lactide) nanoparticles in MPTP-induced parkinsonism. Nanomedicine (Lond.).

[115] Gepner Y., Golan R., Harman-Boehm I., Henkin Y., Schwarzfuchs D., Shelef I., Durst R., Kovsan J., Bolotin A., Leitersdorf E. (2015). Effects of initiating moderate alcohol intake on cardiometabolic risk in adults with type 2 diabetes: A 2-year randomized, controlled trial. Ann. Intern. Med..

[116] Juhasz B., Mukherjee S., Das D.K. (2010). Hormetic response of resveratrol against cardioprotection. Exp. Clin. Cardiol..

[117] Kucinska M., Piotrowska H., Luczak M.W., Mikula-Pietrasik J., Ksiazek K., Wozniak M., Wierzchowski M., Dudka J., Jäger W., Murias M. (2014). Effects of hydroxylated resveratrol analogs on oxidative stress and cancer cells death in human acute T cell leukemia cell line: Prooxidativepotential of hydroxylated resveratrol analogs. Chem. Biol. Interact..

[118] Chang T.K., Chen J., Lee W.B. (2001). Differential inhibition and inactivation of human CYP1 enzymes by trans-resveratrol: Evidence for mechanism-based inactivation of CYP1A2. J. Pharmacol. Exp. Ther..

[119] Chow H.H., Garland L.L., Hsu C.H., Vining D.R., Chew W.M., Miller J.A., Perloff M., Crowell J.A., Alberts D.S. (2010). Resveratrol modulates drug- and carcinogen-metabolizing enzymes in a healthy volunteer study. Cancer Prev. Res. (Phila).

[120] Iwuchukwu O.F., Tallarida R.J., Nagar S. (2011). Resveratrol in combination with other dietary polyphenols concomitantly enhances antiproliferation and UGT1A1 induction in Caco-2 cells. Life Sci..

[121] Detampel P., Beck M., Krähenbühl S., Huwyler J. (2012). Drug interaction potential of resveratrol. Drug Metab. Rev..

[122] Yu C., Shin Y.G., Kosmeder J.W., Pezzuto J.M., van Breemen R.B. (2003). Liquid chromatography/tandemmass spectrometric determination of inhibition of human cytochrome P450 isozymes by resveratrol and resveratrol-3-sulfate. Rapid Commun. Mass Spectrom..

[123] Ogu C.C., Maxa J.L. (2000). Drug interactions due to cytochrome P450. Proceeding (Bayl. Univ. Med. Cent.).

[124] Visioli F. (2014). The resveratrol fiasco. Pharmacol. Res..

[125] Tomé-Carneiro J., Larrosa M., Yáñez-Gascón M.J., Dávalos A., Gil-Zamorano J., Gonzálvez M., García-Almagro F.J., Ros J.A.R., Tomás-Barberán F.A., Espín J.C. (2013). One-year supplementation with a grape extract containing resveratrol modulates inflammatory-related microRNAs and cytokines expression in peripheral blood mononuclear cells of type 2 diabetes and hypertensive patients with coronary artery disease. Pharmacol. Res..

[126] Tang P.C.-T., Ng Y.-F., Ho S., Gyda M., Chan S.-W. (2014). Resveratrol and cardiovascular health—Promising therapeutic or hopeless illusion?. Pharmacol. Res..

[127] Cruz M.N., Luksha L., Logman H., Poston L., Agewall S., Kublickiene K. (2006). Acute responses to phytoestrogens in small arteries from men with coronary heart disease. Am. J. Physiol. Heart Circ. Physiol..

[128] Rakici O., Kiziltepe U., Coskun B., Aslamaci S., Akar F. (2005). Effects of resveratrol on vascular tone and endothelial function of human saphenous vein and internal mammary artery. Int. J. Cardiol..

[129] Amadio P., Baldassarre D., Tarantino E., Zacchi E., Gianellini S., Squellerio I., Amato M., Weksler B.B., Tremoli E., Barbieri S.S. (2015). Production of prostaglandin E2 induced by cigarette smoke modulates tissue factor expression and activity in endothelial cells. FASEB J..

[130] Thushara R.M., Hemshekhar M., Paul M., Sundaram M.S., Shankar R.L., Kemparaju K., Girish K.S. (2014). Crocin prevents sesamol-induced oxidative stress and apoptosis in human platelets. J. Thromb. Thrombolysis.

[131] Zamora-Ros R., Urpí-Sardà M., Lamuela-Raventós R.M., Estruch R., Vázquez-Agell M., Serrano-Martínez M., Jaeger W., Andres-Lacueva C. (2006). Diagnostic performance of urinary resveratrol metabolites as a biomarker of moderate wine consumption. Clin. Chem..

[132] Faghihzadeh F., Adibi P., Hekmatdoost A. (2015). The effects of resveratrol supplementation on cardiovascular risk factors in patients with non-alcoholic fatty liver disease: Arandomised, double-blind, placebo-controlled study. Br. J. Nutr..

[133] Van der Made S.M., Plat J., Mensink R.P. (2015). Resveratrol does not influence metabolic risk markers related to cardiovascular health in overweight and slightly obese subjects: A randomized, placebo-controlled crossover trial. PLoS ONE.

[134] Semba R.D., Ferrucci L., Bartali B., Urpí-Sarda M., Zamora-Ros R., Sun K., Cherubini A., Bandinelli S., Andres-Lacueva C. (2014). Resveratrol levels and all-cause mortality in older community-dwelling adults. JAMA Intern. Med..

[135] Soare A., Weiss E.P., Holloszy J.O., Fontana L. (2014). Multiple dietary supplements do not affect metabolic and cardio-vascular health. Aging (Albany NY).

[136] Huang J.P., Huang S.S., Deng J.Y., Chang C.C., Day Y.J., Hung L.M. (2010). Insulin and resveratrol act synergistically, preventing cardiac dysfunction in diabetes, but the advantage of resveratrol in diabetics with acute heart attack is antagonized by insulin. Free Radic. Biol. Med..

[137] Zhang H., Morgan B., Potter B.J., Ma L., Dellsperger K.C., Ungvari Z., Zhang C. (2010). Resveratrol improves left ventricular diastolic relaxation in type 2 diabetes by inhibiting oxidative/nitrative stress: *In vivo* demonstration with magnetic resonance imaging. Am. J. Physiol. Heart Circ. Physiol..

[138] Barger J.L., Kayo T., Vann J.M., Arias E.B., Wang J., Hacker T.A., Wang Y., Raederstorff D., Morrow J.D., Leeuwenburgh C. (2008). A low dose of dietary resveratrol partially mimics caloric restriction and retards aging parameters in mice. PLoS ONE.

[139] Dang W. (2014). The controversial world of sirtuins. Drug Discov. Today Technol..

[140] Pacholec M., Bleasdale J.E., Chrunyk B., Cunningham D., Flynn D., Garofalo R.S., Griffith D., Griffor M., Loulakis P., Pabst B. (2010). SRT1720, SRT2183, SRT1460, and resveratrol are not direct activators of SIRT1. J. Biol. Chem..

[141] Fernandez-Marcos P.J., Auwerx J. (2011). Regulation of PGC-1α, a nodal regulator of mitochondrial biogenesis. Am. J. Clin. Nutr..

[142] Csiszar A., Labinskyy N., Pinto J.T., Ballabh P., Zhang H., Losonczy G., Pearson K., de Cabo R., Pacher P., Zhang C. (2009). Resveratrol induces mitochondrial biogenesis in endothelial cells. Am. J. Physiol. Heart Circ. Physiol..

[143] Louis X.L., Thandapilly S.J., MohanKumar S.K., Yu L., Taylor C.G., Zahradka P., Netticadan T. (2012). Treatment with low-dose resveratrol reverses cardiac impairment in obese prone but not in obese resistant rats. J. Nutr. Biochem..

[144] Qin F., Siwik D.A., Luptak I., Hou X., Wang L., Higuchi A., Weisbrod R.M., Ouchi N., Tu V.H., Calamaras T.D. (2012). The polyphenols resveratrol and S17834 prevent the structural and functional sequelae of diet-induced metabolic heart disease in mice. Circulation.

[145] Su H.C., Hung L.M., Chen J.K. (2006). Resveratrol, a red wine antioxidant, possesses an insulin-like effect in streptozotocin-induced diabetic rats. Am. J. Physiol. Endocrinol. Metab..

[146] Bagul P.K., Middela H., Matapally S., Padiya R., Bastia T., Madhusudana K., Reddy B.R., Chakravarty S., Banerjee S.K. (2012). Attenuation of insulin resistance, metabolic syndrome and hepatic oxidative stress by resveratrol in fructose-fed rats. Pharmacol. Res..

[147] Turan B., Tuncay E., Vassort G. (2012). Resveratrol and diabetic cardiac function: Focus on recent *in vitro* and *in vivo* studies. J. Bioenerg. Biomembr..

[148] Liu K., Zhou R., Wang B., Mi M.T. (2014). Effect of resveratrol on glucose control and insulin sensitivity: Ameta-analysis of 11 randomized controlled trials. Am. J. Clin. Nutr..

[149] Haohao Z., Guijun Q., Juan Z., Wen K., Lulu C. (2015). Resveratrol improves high-fat diet induced insulin resistance by rebalancing subsarcolemmal mitochondrial oxidation and antioxidantion. J. Physiol. Biochem..

[150] Beaudoin M.S., Perry C.G., Arkell A.M., Chabowski A., Simpson J.A., Wright D.C., Holloway G.P. (2014). Impairments in mitochondrial palmitoyl-CoA respiratory kinetics that precede development of diabetic cardiomyopathy are prevented by resveratrol in ZDF rats. J. Physiol..

[151] Hobbs T., Caso R., McMahon D., Nymark M. (2014). A novel, multi-ingredient supplement to manage elevated blood lipids in patients with no evidence of cardiovascular disease: A pilot study. Altern. Ther. Health Med..

[152] Micans P. (2013). Aortic wave velocity: A noninvasive method to measure the stiffness of arteries and the clinical results of supplements that appear to improve arterial stiffness. Curr. Aging Sci..

[153] Popat R., Plesner T., Davies F., Cook G., Cook M., Elliott P., Jacobson E., Gumbleton T., Oakervee H., Cavenagh J. (2013). A phase 2 study of SRT501 (resveratrol) with bortezomib for patients with relapsed and or refractory multiple myeloma. Br. J. Haematol..

[154] Agarwal B., Campen M.J., Channell M.M., Wherry S.J., Varamini B., Davis J.G., Baur J.A., Smoliga J.M. (2013). Resveratrol for primary prevention of atherosclerosis: Clinical trial evidence for improved gene expression in vascular endothelium. Int. J. Cardiol..

[155] Tomé-Carneiro J., Gonzálvez M., Larrosa M., Yáñez-Gascón M.J., García-Almagro F.J., Ruiz-Ros J.A., García-Conesa M.T., Tomás-Barberán F.A., Espín J.C. (2012). One-year consumption of a grape nutraceutical containing resveratrol improves the inflammatory and fibrinolytic status of patients in primary prevention of cardiovascular disease. Am. J. Cardiol..

[156] Zamora-Ros R., Urpi-Sarda M., Lamuela-Raventós R.M., Martínez-González M.Á., Salas-Salvadó J., Arós F., Fitó M., Lapetra J., Estruch R., Andres-Lacueva C. (2012). PREDIMED study investigators. High urinary levels of resveratrol metabolites are associated with a reduction in the prevalence of cardiovascular risk factors in high-risk patients. Pharmacol. Res..

[157] Fujitaka K., Otani H., Jo F., Jo H., Nomura E., Iwasaki M., Nishikawa M., Iwasaka T., Das D.K. (2011). Modified resveratrol Longevinex improves endothelial function in adults with metabolic syndrome receiving standard treatment. Nutr. Res..

[158] Wong R.H., Howe P.R., Buckley J.D., Coates A.M., Kunz I., Berry N.M. (2011). Acute resveratrol supplementation improves flow-mediated dilatation in overweight/obese individuals with mildly elevated blood pressure. Nutr. Metab. Cardiovasc. Dis..

[159] Karlsen A., Paur I., Bøhn S.K., Sakhi A.K., Borge G.I., Serafini M., Erlund I., Laake P., Tonstad S., Blomhoff R. (2010). Bilberry juice modulates plasma concentration of NF-kappaB related inflammatory markers in subjects at increased risk of CVD. Eur. J. Nutr..

[160] Gresele P., Pignatelli P., Guglielmini G., Carnevale R., Mezzasoma A.M., Ghiselli A., Momi S., Violi F. (2008). Resveratrol, at concentrations attainable with moderate wine consumption, stimulates human platelet nitric oxide production. J. Nutr..

[161] Lekakis J., Rallidis L.S., Andreadou I., Vamvakou G., Kazantzoglou G., Magiatis P., Skaltsounis A.L., Kremastinos D.T. (2005). Polyphenolic compounds from red grapes acutely improve endothelial function in patients with coronary heart disease. Eur. J. Cardiovasc. Prev. Rehabil..

